# The influence of scuba diving experience on divers’ perceptions, and its implications for managing diving destinations

**DOI:** 10.1371/journal.pone.0219306

**Published:** 2019-07-05

**Authors:** Serena Lucrezi, Martina Milanese, Carlo Cerrano, Marco Palma

**Affiliations:** 1 TREES – Tourism Research in Economics, Environs and Society, North-West University, Potchefstroom, South Africa; 2 Studio Associato GAIA s.n.c., Genova, Italy; 3 Department of Life and Environmental Sciences (DiSVA), Polytechnic University of Marche, UO CoNISMa, Via Brecce Bianche, Ancona, Italy; 4 UBICA s.r.l., Genova, Italy; University of Pavia, ITALY

## Abstract

Scuba diving experience–which can include accumulated diving experience and familiarity with a diving location–is an important descriptor of diver specialisation and behaviour. Formulating and applying generalisations on scuba diving experience and its effects could assist the management of diving destinations around the world. This requires research that tests whether the influences of scuba diving experience are consistent across divers’ segments at different locations. The study assessed and compared the influence of scuba diving experience at two study areas in Italy and Mozambique. Scuba divers (N = 499) participated in a survey of diver segmentation, experience, and perceptions. The influence of diving experience on perceptions was determined using canonical correspondence analysis (CCA). Experienced divers provided positive self-assessments, were less satisfied with dive sites’ health and management, and viewed the impacts of scuba diving activities less critically than novice divers. Scuba diving experience exerted similar influences on divers, regardless of the study area. However, remarkable differences also emerged between the study areas. Therefore, the use of generalisations on scuba diving experience remains a delicate issue. Recommendations were formulated for the management of experienced scuba diving markets and for the use of generalisations on diving experience to manage diving destinations.

## Introduction

### Scuba diving segmentation research and the role of experience

Scuba diving is an activity characterised by an important recreational component as well as a professional component including commercial and scientific diving. Recreational scuba diving, in particular, is a leisure and tourism sector affecting the communities, economies and environments of destinations across all latitudes in both developed and developing countries [1]. In marine protected areas (MPAs), for example, scuba diving is a recreational activity which, when properly regulated, can generate income for MPA management and for communities surrounding MPAs [2–3]. On the other hand, uncontrolled scuba diving can pose serious threats to the conservation agendas of MPAs through either direct (e.g. contact) or indirect (e.g. pollution) impacts [4–5].

Segmentation research at diving destinations has been central to the understanding of the general profile of scuba divers for the purpose of marketing, management and sustainable development planning [6]. In this context, scuba divers have also been categorised according to their level of experience (S1 Table). Investigating how experience influences the way scuba divers think and act can be critical in formulating strategies to manage diving tourism, diving activities and marine environments. Scuba diving experience can affect divers’ motivations, preferences, attitudes, underwater actions, and perceptions of the quality of the environments in which they dive [7].

As the diversification (according to the level of experience) of the scuba-diving tourism market is likely to affect all diving destinations indiscriminately, the influence and implications of scuba diving experience could be considered a constant factor across divers’ segments at various destinations. Consequently, generalisations regarding scuba diving experience could be formulated and applied to different markets and diving destinations worldwide for management purposes. However, pooled research assessing the influence of scuba diving experience in different contexts shows contrasting results, with scuba diving experience affecting divers’ attitudes, perceptions and behaviour either positively or negatively [5,8–9].

These contradictions, which make it difficult to understand whether experience exerts similar influences on different groups of divers and at different destinations, have consequences for both research and management. One reason behind the contrasting results is that scuba diving experience has been measured through different sets of variables, since it can be defined in many ways. Comparing the influence of a given construct that defines scuba diving experience across typologies of divers and locations can possibly help to elucidate documented contrasts. To date, almost no research has experimented with such a system.

### Defining scuba diving experience and its influence

Much research has categorised divers’ experience levels according to the number of years diving and the highest scuba diving qualification held (S1 Table). In time, however, the need to deploy more variables to explain scuba diving experience has become evident [10–14]. Research has been using scuba diving experience as one of the components characterising scuba diver specialisation (S1 Table). Scuba diving specialisation, a multidimensional index that includes behavioural, cognitive and affective (conative) domains, has successfully predicted scuba divers’ perceptions, attitudes and behaviour [12,15]. However, if represented by the right variables, accumulated scuba diving experience, which includes aspects in the three domains of diver specialisation, remains a valid tool to segment and study scuba divers [11,16–17]. Accumulated scuba diving experience should at least include indicators of diving history, for example the total number of years diving and the total number of logged dives; indicators of regular practice and commitment, for example annual diving frequency and time elapsed since the last dive; and indicators of development, for example certification level.

Accumulated scuba diving experience is viewed as the primary element shaping the growth of scuba divers. The transformation from a novice to an experienced diver is generally accompanied by the improvement of skills and a shift in motivations to dive, expectations of the diving experience, preferences, satisfaction, attitudes towards conservation and management, and behaviours in and out of the water [7,15,18–20]. In addition, the development of divers is normally underpinned by their growing attachment to the diving activity, the importance of the resources the diving activity depends on, for example environment and safety, and the desire to improve and to learn [12,21–23]. Thus, accumulated scuba diving experience can play a crucial role in affecting diving activities and informing the management of diving tourism and destinations (S2 Table).

Familiarity with dive locations is also an indicator of scuba diving experience and specialisation (S1 Table). A number of variables can be ascribed to familiarity with a dive location, for example the number of dives logged, the number of years diving, and annual diving frequency at that location. Studies looking at the influences of familiarity with dive locations on divers have yielded mixed results, impinging on management guidelines. On the one hand, scuba divers who are familiar with a dive location tend to become attached to it and thus willing to either deepen their knowledge about its biological characteristics or pay for its conservation [22,24]. Loyal divers also tend to have more realistic expectations of ecosystem conditions, for example coral cover and fish abundance, and thus tend to be satisfied with their experience [22,25]. On the other hand, scuba divers who are attached to a particular location can become intolerant of changes such as an increasing influx of tourists, crowding of dive sites and the introduction of man-made structures [26]. Despite the attachment to a diving destination, experienced divers can be prepared to abandon it when unacceptable levels of degradation are perceived [27]. Given these influences, familiarity deserves special attention in scuba diving research and can be a valuable addition to variables representing scuba diving experience [26,28].

### Aim of the study

The aim of this study was to test the influence of scuba diving experience, underlain by a given group of variables, on divers’ perceptions relevant to environmental, destination, and business management across two study areas. The choice of study areas fell on destinations beyond the tropics. This choice was grounded on two considerations. First, research on scuba diving tourism at destinations beyond the tropics is still limited [29]. Second, based on the relatively few available studies (compared with the literature available for tropical destinations), locations beyond the tropics tend to host a good proportion of experienced divers [30–33]. For this study, we selected one diving destination in the northern hemisphere that is further from the tropic (temperate climate), and one in the southern hemisphere that is closer to the tropic (subtropical climate). The selected locations present some notable differences in climate, environmental conditions, local history of diving activities, the type of local diving activities and attractions, and the average diver profile. Both study areas share the status of being protected areas. Protected areas such as MPAs and marine reserves tend to attract scuba diving tourism [3]. They are subjected to regulations that are likely to affect diving activities [2,15]. And they can set an example for destinations that are not officially protected and are heavily affected by the impacts of human activities, including scuba diving [5,14,34].

The following research questions were formulated for this study: Does diving experience influence divers’ perceptions? Is this influence similar across different study areas? Answering these questions will have implications for the management of diving tourism based on market segmentation, and for the formulation and application of generalisations on diving experience to diving destinations. Such implications will be relevant for both authorities and local businesses.

## Materials and methods

This study was approved by the Faculty of Economic and Management Sciences Research Ethics Committee (EMS-REC) at the North-West University under the ethics code EMS2016/11/25-0237. No private personal information was asked from the participants in the study. The data were handled according to laws on privacy and oral consent was provided by the participants before the study. Participants were able to leave the research at any point during the study.

### Study areas

**Fig 1:**
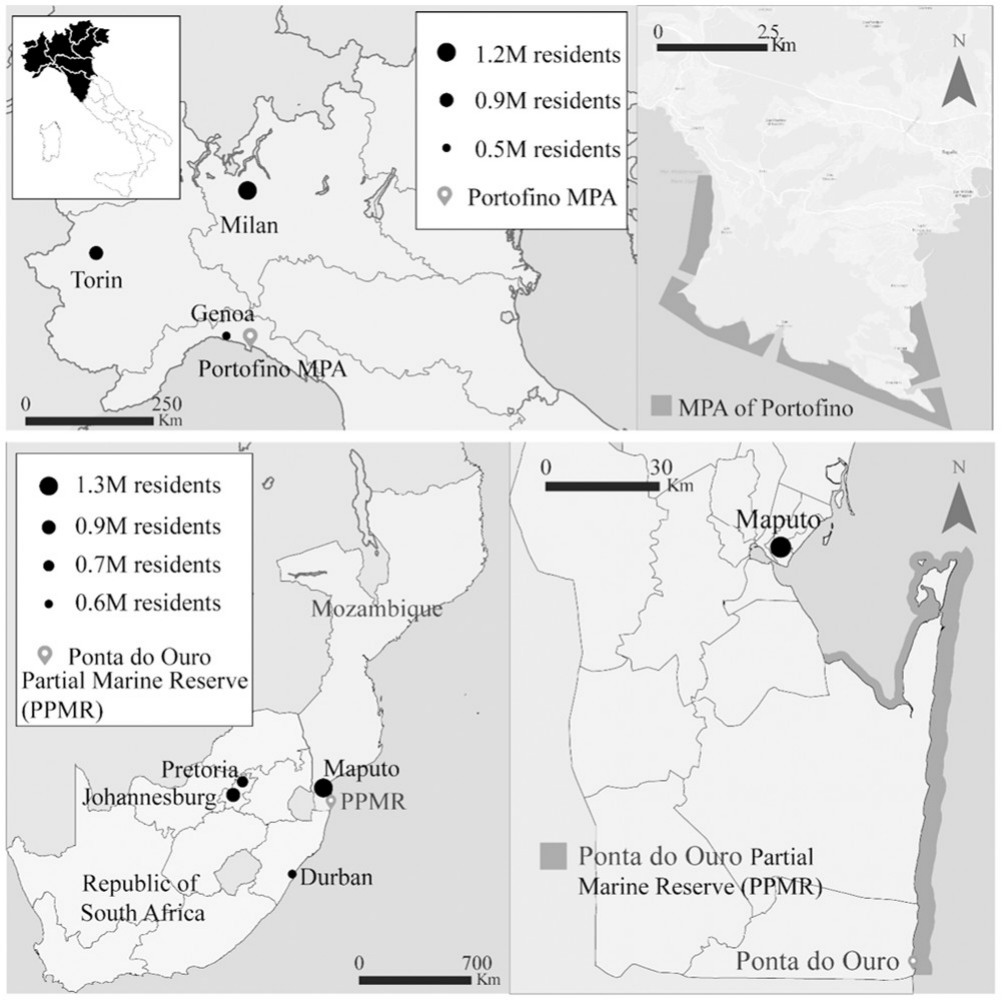
Geographic location of the study areas, namely the Portofino Marine Protected Area (MPA) in northwestern Italy, and Ponta do Ouro in the Ponta do Ouro Partial Marine Reserve (PPMR), southern Mozambique.

Two study areas were selected for the research, specifically the Portofino MPA in northern Italy, and the Ponta do Ouro Partial Marine Reserve (PPMR) in southern Mozambique (Fig 1). Large inland cities in the range of 215 km from Portofino and 645 km from Ponta do Ouro (Fig 1) provide the majority of the scuba diving clientele, which is mostly characterised by daily visitors in the case of Italy, and overnight stayers in the case of Mozambique [35].

The Portofino MPA (with an area of 3.74 km^2^) is located in a small but populated area of the region Liguria in the northwestern Mediterranean Sea (Fig 1). The area has a temperate climate, with annual sea surface temperatures (SST) averaging 18 °C [36]. The MPA was established in 1999 [37], yet scuba diving tourism has been present in the area for several decades [35]. Considering trends from 2012 until today, the average number of dives recorded in the MPA annually is about 50,000 [35,38]. The prominent underwater habitats in the MPA include seagrass meadows, vertical rocky walls of Oligocenic pudding stone, coralligenous biocenoses, boulders and stones, caves and caverns, and the pelagic zone.

Since its establishment, the MPA has achieved important goals, for example increasing fish biomass, protecting endangered species such as the grouper and red coral, and safeguarding ecosystems such as seagrass meadows [39–40]. The MPA is zoned to control uses, including artisanal fishing, scuba diving, free diving, snorkelling, and boating. Given the small area of the MPA and the many uses, some of which tend to overlap, conflicts between stakeholders such as divers and fishermen can occur [35,41].

Scuba diving activities in the MPA are strictly regulated and limited to a number of dive sites marked by fixed mooring buoys. A study dating back to 2005 described the scuba diving population of Portofino as male-dominated, moderately experienced (generally at divemaster level) and loyal to the destination (visiting the destination up to 30 times per year) [42]. The peak diving season in the MPA is from June to September (summer season) [35], although an off-season market also exists which focuses on technical dives and training dives (S. Lucrezi, pers. obs.). The dives offered in the MPA are both recreational, following established routes along vertical rocky walls and reaching depths of up to 40 m, and technical and wreck dives, which can exceed recreational depths [43].

A famous shark diving destination, Ponta do Ouro is a small village at the southern end of the PPMR (with an area of 678 km^2^) and close to the border between Mozambique and South Africa (Fig 1). The area has a subtropical climate, with SST averaging 25 °C [44]. Scuba diving activities in Ponta do Ouro are characterised by a busy season from December to April, alternated with a quieter season during the rest of the year [45]. Approximately 30,000 dives are logged from the launching site in Ponta do Ouro annually, although the total number of dives logged for the entire PPMR is greater [46].

The PPMR was proclaimed in 2009, but it was already a popular scuba diving destination before its establishment [47–48]. Various uses are allowed in the multiple-use zone of the PPMR where Ponta do Ouro is located. These include snorkelling, surfing, swimming with dolphins, recreational (sport) fishing, and scuba diving on sandstone reefs and outcrops largely populated by soft coral [49]. The establishment of the PPMR has allowed the regulation of scuba diving activities, which were previously threatening the underwater ecosystems due to uncontrolled crowding of dive sites [50]. However, important construction threats and impacts from abusive fishing and littering persist to this day [35,45,51]. Research dating back to 2003 described the scuba diving population of Ponta do Ouro as dominated by male newcomers with approximately six years of scuba diving history and an average of 50 logged dives [50].

### Research design and data collection

The research followed a quantitative, descriptive and non-experimental design, with a structured questionnaire survey being the instrument of data collection [S3 Table]. In this study, scuba diving experience was defined by two sets of variables, namely accumulated diving experience and familiarity with diving at the study areas. Divers’ perceptions were defined by three sets of variables, namely divers’ self-assessment, satisfaction with the diving at the study areas, and perceptions of scuba diving impacts on the environment.

The first section in the questionnaire included questions on demography (gender, age, education, country of residence, marital status and occupation), accumulated diving experience and familiarity with the study areas, and asked the divers whether they engaged in underwater photography at the destination (by means of a Yes/No question).

Accumulated diving experience was defined by four variables. The first variable was the total number of scuba diving certifications held (whatever the certifying agency) under the following categories: basic (from the equivalent of PADI Open Water Diver to that of PADI Rescue), professional (from the equivalent of PADI Divemaster to Instructor, all levels), specialties (such as Enriched Air Diver, Deep Diver, Wreck Diver and Dry Suit Diver), technical (such as Advanced Nitrox, Trimix, Cave and Deco), and dry (such as Oxygen Provider and Gas Blender). The second variable was the total number of years diving. The third variable was the total number of dives logged. The fourth variable was the average number of dives logged annually. Familiarity with the study areas was expressed as the total number of dives logged at the study areas, and the average number of dives logged at the study areas annually.

The second section in the questionnaire asked scuba divers to assess themselves by using a five-point Likert scale of agreement with a number of statements (where 1 = strongly disagree and 5 = strongly agree). Statements included aspects of knowledge (e.g., *I know all the local diving regulations*), environmental attitudes (e.g., *I would like to be involved in local marine conservation*) and behaviour (e.g., *I keep a good distance from the bottom habitats when I dive*), particularly in the context of the study areas. Previous research demonstrated that self-reported behaviour of divers can be used as a valuable substitute for observed behaviour [9,52–53]. Therefore, self-reported behaviour was seen as an indication of actual diver behaviour.

The third section in the questionnaire included a set of three-point Likert scale questions on satisfaction (where 1 = unsatisfied and 3 = satisfied) with the diving sites at the study areas, particularly with reference to environmental (e.g., underwater cleanliness and visibility, general health of the dive sites, variety of species) and management aspects (e.g., crowding of dive sites, pre-dive briefing, litter).

The fourth and last section invited the divers to rate the potential ecological damage caused by diving-related actions by using a four-point Likert scale (where 1 = no damage and 4 = heavy damage). Some of these actions included anchoring, underwater photography, intentional and unintentional contact with mobile and sessile wildlife, diving after drinking alcohol, and a poor pre-dive briefing.

The population under investigation were the scuba divers visiting the study areas. Data were collected during the summer months of 2015 and 2016. This period corresponds to peak diving at the study areas [35]. Using the recorded number of dives logged at the study areas annually (approximately 50,000 in Portofino and 30,000 in Ponta do Ouro) and assuming that a single diver would log at least three dives per year at each study area [10], the population was established as approximately 17,000 divers in Portofino and 10,000 in Ponta do Ouro.

Of these populations, a sample of 370 scuba divers would yield a confidence level of 95% and a margin of error of 5% [54]. Thus, a total of 400 questionnaires were printed for each study area. All 400 questionnaires were distributed in Portofino, of which 279 were completed and returned, yielding a participation of 70%. In Ponta do Ouro, only 300 questionnaires were distributed due to the poor turnover of divers during the time of sampling. A total of 220 questionnaires were completed and returned, yielding a participation of 73%. The number of questionnaires that had successfully been completed guaranteed an acceptable sample size at both study areas (a 95% confidence level and 6% margin of error).

Sampling was done every second day for a total of 30 days per study area. The fieldworkers collected data at most dive centres that operated at each study area. On each sampling day, two fieldworkers visited either one or two dive centres and haphazardly invited scuba divers returning from a dive to participate in the questionnaire survey, which took ten minutes to complete. Divers were invited to remain at the dive centre while completing the questionnaire; this ensured that questionnaires were completed and returned to the fieldworkers in time.

### Data analysis

The demographic and experience profiles of the scuba divers were determined through descriptive statistics, frequency tables and breakdown statistics to compare variables across study areas in a descriptive manner. Binomial logistic regressions were used to test whether associations existed between accumulated scuba diving experience and the use of underwater cameras, which is normally associated with specialised scuba diving as well as negative ecological impacts to bottom habitats [5,8].

Following normality (chi-squared) tests, either a one-way analysis of variance (ANOVA) or a Mann-Whitney U test was performed to highlight significant differences in divers’ demography and experience between study areas. The magnitude of the differences between study areas was determined by calculating practical effect size (Cohen’s *d*) [55]. Cohen suggested that *d* = 0.2 represents a low, *d* = 0.5 a medium and *d* = 0.8 a large effect size.

Two exploratory analyses were performed on divers’ perceptions (self-assessment, satisfaction with diving at the study areas, and perceptions of scuba diving impacts). The first one is the exploratory factor analysis (EFA), employed to reduce the size of a dataset by identifying relationships between questionnaire items and extracting latent factors (determined by eigen values and factor loadings as cut-off criteria, and then calculated as factor scores) underlying the items [56]. The second one is the reliability test, which checks for internal consistency (Cronbach’s α value) of the factors extracted through EFA [57]. The means of factor scores between study areas were compared through a general linear model (analysis of covariance [ANCOVA]) in which all the significantly different demographic and experience variables between study areas were included as co-variates. The magnitude of the differences between the study areas was determined by calculating the practical effect size (Cohen’s *d*). Correlational relationships between variables of accumulated diving experience, familiarity with the study areas, and factor scores were investigated using the nonparametric Spearman’s rank-order correlations (*r*_s_). All above analyses were performed using the Statsoft Statistica software, Version 13.2 (2016).

The influence of divers’ scuba diving experience (accumulated diving experience and familiarity with the study areas) on their perceptions was determined using a canonical correspondence analysis (CCA), a constrained ordination method using correspondence analysis [58]. This multivariate technique was developed in ecology to investigate the abundance of species in relation to environmental variables; however, it is also deployed in other domains such as the social and economic sciences [59]. The main result of CCA is the ordination of the principal dimensions of the dependent variables (points) in a bi-dimensional space, determined by two axes and constrained by the explanatory variables (vectors). Two CCAs were performed, one testing the influence of accumulated diving experience and the other the influence of familiarity with the study areas. The CCAs were performed with the PAST statistical software, Version 2.17 [60], following the eigenanalysis algorithm of Legendre and Legendre [61].

## Results

### Profile of the scuba divers

The variables of demography and the participants’ scuba diving experience, as well as the statistically significant differences in these variables between study areas, are displayed in Table 1. The proportion of male participants in Portofino was greater (79%), whereas Ponta do Ouro had a similar ratio of males to females (55% to 45%). The divers were either in their late 30s (Ponta do Ouro) or in their early 40s (Portofino), with a moderate (Ponta do Ouro) to high (Portofino) level of education. Divers were primarily Italian in Portofino (82%) and South African in Ponta do Ouro (69%). Other markets were mostly European for both study areas. Most divers were either single or married and had paid employment, regardless of the study area.

**Table 1:** Demographic and experience profile of the scuba divers participating in the study (N = 499).

Variable	Portofino	Ponta do Ouro	Differences between study areas
Gender	Male (79%); female (19%)	Male (55%); female (45%)	(A): MS _(1,491)_ = 7.91; F = 39.81*** Cohen’s *d* = 0.55 (moderate)
Age	Range: 15–70; mean ± SD: 42.9 ± 11.6; SE: 0.7	Range: 16–67; mean ± SD: 39.5 ± 13; SE: 0.9	(A): MS _(1,469)_ = 1378.7; F = 9.23** Cohen’s *d* = 0.28 (low)
Education level	Undergraduate (43%); postgraduate (38%); high school diploma (16%); other (3%)	High school diploma (49%); undergraduate (30%); postgraduate (19%); other (2%)	(A): MS _(1,494)_ = 68.32; F = 68.65*** Cohen’s *d* = 0.71 (large)
Country of residence	Italy (82%); Europe (16%); other (2%)	South Africa (69%); Europe (22%); other (9%)	--
Marital status	Single (40%); married (37%); other (23%)	Single (41%); married (41%); other (18%)	--
Occupation	Paid work (78%); student (7%); other (15%)	Paid work (75%); student (14%); other (11%)	--
Pooled number of SDC^#^	Range: 1–45; mean ± SD: 6.9 ± 7; SE: 0.4	Range: 1–39; mean ± SD: 4.3 ± 6; SE: 0.4	(U): Z adjusted = 7.8*** Cohen’s *d* = 0.4 (moderate)
Number of basic SDC	Range: 1–23; mean ± SD: 2.7 ± 1.6; SE: 0.1	Range: 1–10; mean ± SD: 2 ± 1.2; SE: 0.1	(U): Z adjusted = 7.57*** Cohen’s *d* = 0.49 (moderate)
Number of professional SDC	Range: 1–20; mean ± SD: 2.9 ± 3.1; SE: 0.3	Range: 1–17; mean ± SD: 2.6 ± 3; SE: 0.4	(U): Z adjusted = 0.76^ns^ Cohen’s *d* = 0.1 (low)
Number of specialty SDC	Range: 1–20; mean ± SD: 3.1 ± 3.2; SE: 0.2	Range: 1–20; mean ± SD: 3.4 ± 4.8; SE: 0.6	(U): Z adjusted = 1.79^ns^ Cohen’s *d* = 0.1 (low)
Number of technical SDC	Range: 1–11; mean ± SD: 2.6 ± 2.2; SE: 0.3	Range: 1–5; mean ± SD: 2 ± 1.3; SE: 0.3	(U): Z adjusted = 0.51^ns^ Cohen’s *d* = 0.33 (low)
Number of dry SDC	Range: 1–6; mean ± SD: 1.7 ± 1.1; SE: 0.1	Range: 1–10; mean ± SD: 1.9 ± 1.7; SE: 0.3	(U): Z adjusted = -0.32^ns^ Cohen’s *d* = 0.14 (low)
Number of years diving	Range: 1–49; mean ± SD: 12 ± 9.8; SE: 0.6	Range: 1–42; mean ± SD: 10 ± 9.2; SE: 0.6	(U): Z adjusted = 2.34* Cohen’s *d* = 0.21 (low)
Number of dives logged	Range: 1–5000; mean ± SD: 383 ± 705; SE: 43	Range: 1–4700; mean ± SD: 336 ± 375; SE: 51	(U): Z adjusted = 7.77*** Cohen’s *d* = 0.08 (low)
Number of dives per year	Range: 1–500; mean ± SD: 41 ± 61; SE: 3.8	Range: 1–400; mean ± SD: 37 ± 60; SE: 4.4	(U): Z adjusted = 3.63*** Cohen’s *d* = 0.07 (low)
Number of dives logged at study area	Range: 1–3800; mean ± SD: 142 ± 374; SE: 23	Range: 1–2000; mean ± SD: 69 ± 191; SE: 13	(U): Z adjusted = 4.02*** Cohen’s *d* = 0.25 (low)
Number of dives per year at study area	Range: 0^a^ -300; mean ± SD: 21 ± 41; SE: 2.6	Range: 0^a^ -52; mean ± SD: 2.3 ± 6; SE: 0.4	(U): Z adjusted = 9.91*** Cohen’s *d* = 0.64 (moderate)

^#^ Scuba diving certifications.

^a^ Denotes people who were either visiting the study area for the first time or were not planning to visit the study area in the future.

^ns^ Non-significant

* *p* < 0.05

** *p* < 0.01

*** *p* < 0.001

(A) ANOVA; (U) Mann-Whitney U.

The participants varied from novice to professional divers (Table 1). Participants in Portofino held a mean of seven scuba diving certifications, while divers in Ponta do Ouro possessed a mean of over four scuba diving certifications. The divers tended to be certified with PADI (57% in Ponta do Ouro and 43% in Portofino), although as many as 28 other agencies were mentioned by divers in Portofino and eight in Ponta do Ouro. The participants had been diving for a period of one to 49 years, with a mean of 12 years in Portofino and 10 years in Ponta do Ouro (Table 1). They had logged between a single dive and 5,000 dives, with a mean of around 380 for Portofino and 335 for Ponta do Ouro. They logged between one and 500 dives annually, with a mean of 41 for Portofino and 37 for Ponta do Ouro (Table 1).

The familiarity of the participants with the study areas ranged from *none* (a single dive at the study areas and no annual visits) to *very good* (thousands of dives logged at the study areas with hundreds of dives logged there annually); it is thus evident that some of the participants had done the majority of their diving at the study areas (Table 1). However, the mean number of dives logged in Portofino was more than twice the number in Ponta do Ouro, and the mean number of dives logged annually in Portofino was nine times greater than in Ponta do Ouro (Table 1).

Less than half (43%) of the participants in Portofino and half of those in Ponta do Ouro declared that they used underwater cameras while scuba diving. Results from a binomial logistic regression show that two experience variables, namely the number of scuba diving certifications held and the number of dives logged, had a significant positive association with underwater photography (Wald test statistics _number SDC_ = 11.92, *p* < 0.001; _number dives logged_ = 8.82, *p* = 0.03).

### Divers’ perceptions

Descriptive statistics for items used in scaled data are included in S4 Table. The exploratory factor analyses (EFA) performed on divers’ perceptions (self-assessment, satisfaction with diving at the study areas, and perceptions of scuba diving impacts) yielded a total of eight factors (Table 2). Items characterising each factor (also listed in Table 2) had loadings exceeding the cut-off value of 0.40 [56]. All factors were reliable, with Cronbach’s α values above the threshold of 0.60 [57]. The self-assessment of divers was characterised by an assessment of personal *knowledge of local conservation*, personal *underwater skills and behaviour*, and *attitudes towards conservation*. Satisfaction with diving at the study areas was divided into two factors, namely satisfaction with the *ecosystem health* of the dive sites and with the local *management*. Perceptions of scuba diving impacts were divided into perceptions of the damage caused by *direct impacts*, *contingent impacts*, and *irresponsible actions*.

**Table 2:** Descriptive statistics for factors (F) extracted through exploratory factor analysis (EFA) and reliability tests under the following categories: Divers’ self-assessment; divers’ satisfaction with diving at the study areas; and divers’ perceptions of scuba diving impacts.

Source / Likert scaling	Mean ± SD, SE (All)	Mean ± SD, SE (Portofino)	Mean ± SD, SE (Ponta do Ouro)	Factor loadings	Eigen-value	Variance explained (%)	Cronbach alpha (α)
Divers’ self-assessment on a scale where 1 = strongly disagree and 5 = strongly agree							
*F1*: *Knowledge of local conservation*	3.54 ± 0.92, 0.04	3.8 ± 0.88, 0.05	3.2 ± 0.86, 0.06	0.72–0.79	5.39	38.5	0.81
ANCOVA MS _(1,354)_ = 11.91; F = 16.17***; Cohen’s *d* = 0.69 (moderate)							
Items: I know all diving regulations locally; I know the marine conservation programmes that are run locally; I know what fines are given for breaking diving regulations locally; I know what pre-diving procedures have to be followed locally.							
*F2*: *Underwater skills and behaviour*	4.25 ± 0.58, 0.03	4.3 ± 0.57, 0.03	4.19 ± 0.59, 0.04	0.55–0.75	1.77	12.7	0.82
ANCOVA MS _(1,253)_ = 0.07; F = 0.25^ns^; Cohen’s *d* = 0.19 (low)							
Items: I keep neutrally buoyant at all times while diving; I keep a good distance from the bottom habitats while diving; I possess the necessary skills to dive locally; I know how to use scuba diving equipment; I practice good finning technique when I dive; I observe wildlife quietly without chasing it when I dive.							
*F3*: *Attitudes towards conservation*	3.62 ± 0.87, 0.04	3.72 ± 0.82, 0.05	3.5 ± 0.91, 0.06	0.70–0.76	1.31	9.3	0.79
ANCOVA MS _(1,353)_ = 0.31; F = 0.41^ns^; Cohen’s *d* = 0.25 (low)							
Items: I reproach divers who do not pay attention to the pre-dive briefings; I am or want to be involved in local marine conservation; I would like to be involved in local conservation; I reproach divers who break underwater rules.							
Divers’ satisfaction with diving at the study areas on a scale from 1 = unsatisfied to 3 = satisfied							
*F1*: *Ecosystem health*	2.60 ± 0.36, 0.02	2.63 ± 0.32, 0.02	2.56 ± 0.4, 0.03	0.40–0.77	3.91	30.1	0.74
ANCOVA MS _(1,348)_ = 0.42; F = 3.58^ns^; Cohen’s *d* = 0.19 (low)							
Items: Water cleanliness; abundance of marine life; visibility; variety of small species; variety of coral and sessile life; the general health of the dive sites; variety of big species							
*F2*: *Management*	2.50 ± 0.41, 0.02	2.53 ± 0.38, 0.02	2.46 ± 0.44, 0.03	0.40–0.78	1.71	13.1	0.73
ANCOVA MS _(1,348)_ = 1.81; F = 11.62***; Cohen’s *d* = 0.17 (low)							
Items: Litter; local diving regulations; crowding of dive sites; the underwater conduct of fellow divers; the pre-dive briefing; the conduct of the divemaster							
Divers’ perceptions of scuba diving impacts on a scale, ranging from 1 = no damage to 4 = heavy damage							
*F1*: *Direct impacts*	3.11 ± 0.67, 0.03	3.38 ± 0.49, 0.03	2.75 ± 0.7, 0.05	0.48–0.77	6.44	26.8	0.83
ANCOVA MS _(1,355)_ = 21; F = 58.58***; Cohen’s *d* = 1.04 (large)							
Items: Walking on the sandy bottom before or during a dive; purposefully touching mobile wildlife; collecting shells, pieces of coral or other; spear fishing; anchoring the boat before a dive; purposefully touching sessile wildlife; touching sessile wildlife accidentally; touching mobile wildlife accidentally							
*F2*: *Contingent impacts*	2.09 ± 0.52, 0.02	2.13 ± 0.51, 0.03	2.05 ± 0.54, 0.04	0.42–0.76	2.66	11.1	0.77
ANCOVA MS _(1,356)_ = 0.13; F = 0.53^ns^; Cohen’s *d* = 0.15 (low)							
Items: Diving with gloves; using flash photography; being a novice diver; drift diving; videotaping underwater; diving at night; wearing sunscreen before a dive; achieving buoyancy near or on the bottom; noise from boats on the surface; using special equipment/configurations							
*F3*: *Irresponsible actions*	2.82 ± 0.74, 0.03	2.9 ± 0.68, 0.04	2.72 ± 0.81, 0.06	0.55–0.75	1.68	7	0.8
ANCOVA MS _(1,356)_ = 1.62; F = 2.89 ^ns^; Cohen’s *d* = 0.14 (low)							
Items: Diving alone and not with a buddy/group; a bad pre-dive briefing; losing or leaving gear behind underwater; not diving with an operator or charter; drinking and diving; chasing or standing in the way of mobile wildlife							

^ns^ Non-significant

*** *p* < 0.001.

Scuba divers at both study areas were confident in their own underwater skills and behaviour and tended to have positive attitudes towards conservation (Table 2). Divers in Portofino believed that they possessed good knowledge of local conservation initiatives, whereas those in Ponta do Ouro believed to possess average knowledge (Table 2). Scuba divers were neutral to satisfied with diving at the study areas, although satisfaction with local management was slightly higher in Portofino than in Ponta do Ouro, where litter management received a relatively low score compared with other items (Table 2).

Divers at both study areas believed that contingent impacts by divers were small and caused minimal damage to underwater ecosystems, whereas direct impacts and irresponsible actions caused moderate damage (Table 2). Divers in Portofino saw the damage of direct impacts as significantly greater compared with divers in Ponta do Ouro, where items including walking on sand before or during a dive, touching mobile wildlife accidentally, and collecting shells received low scores for damage (Table 2).

### Influence of scuba diving experience on divers’ perceptions

**Fig 2:**
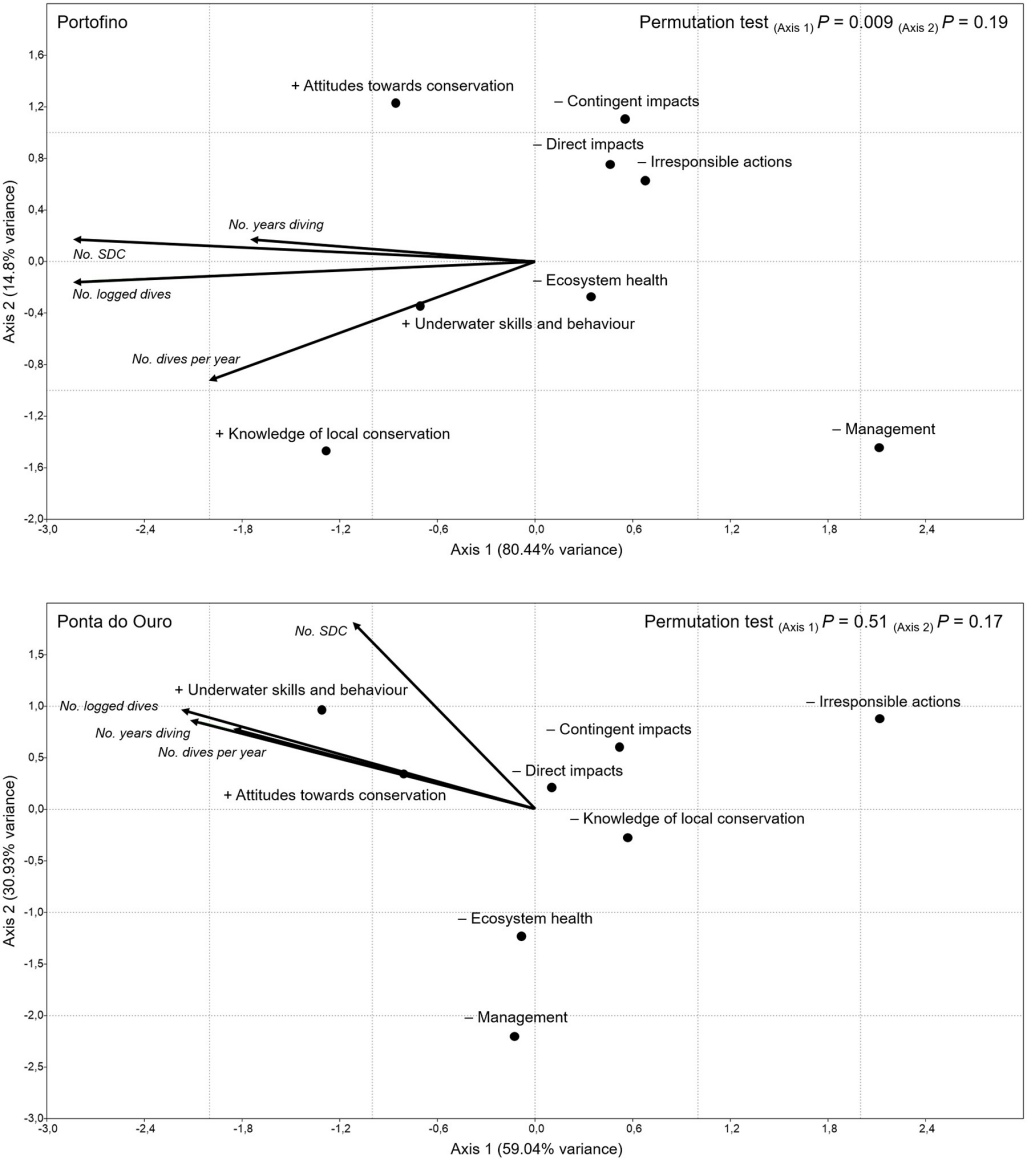
Canonical correspondence analysis (CCA) ordination biplot, displaying the influence of scuba diving experience (vectors) on factors of divers’ self-assessment, satisfaction with diving at the study areas, and perceptions of scuba diving impacts (points) at the study areas. The “+” symbol next to a factor represents a positive influence, whereas the “˗” symbol represents a negative influence.

The results of the canonical correspondence analysis (CCA), assessing the influence of accumulated diving experience on divers’ perceptions for both study areas, are displayed in Fig 2. Variables describing accumulated diving experience were positively correlated (Table 3). Axis 1 and Axis 2 in the ordination biplot (Fig 2) accounted for nearly all the variance in the data; thus, the variation in perceptions was well predicted by accumulated diving experience.

**Table 3:** Nonparametric Spearman rank order correlations (*r*_s_) between variables used in the canonical correspondence analysis (CCA).

		1	2	3	4	5	6	7	8	9	10	11	12	13	14
1	Number of SDC	1.00													
2	Number of years diving	**0.47**	1.00												
3	Number of logged dives	**0.72**	**0.76**	1.00											
4	Number of dives per year	**0.68**	**0.34**	**0.80**	1.00										
5	Number of dives logged at study area	**0.55**	**0.48**	**0.68**	**0.52**	1.00									
6	Number of dives per year at study area	**0.53**	**0.29**	**0.48**	**0.46**	**0.82**	1.00								
7	Knowledge of local conservation	0.27	0.10	0.21	0.18	**0.39**	**0.50**	1.00							
8	Underwater skills and behaviour	**0.38**	**0.31**	**0.41**	**0.38**	**0.40**	**0.36**	**0.52**	1.00						
9	Attitudes towards conservation	0.17	0.12	0.15	0.13	0.21	0.23	**0.50**	**0.44**	1.00					
10	Ecosystem health	0.03	-0.02	0.07	0.04	0.19	0.20	0.27	0.19	0.10	1.00				
11	Management	-0.15	-0.12	-0.15	-0.11	-0.11	-0.05	0.08	-0.05	-0.02	**0.40**	1.00			
12	Direct impacts	0.24	0.05	0.10	0.13	0.06	0.21	0.25	0.21	0.24	0.03	0.01	1.00		
13	Contingent impacts	0.08	0.07	0.06	0.03	-0.01	0.01	0.13	0.03	0.21	-0.03	-0.10	**0.34**	1.00	
14	Irresponsible actions	-0.03	-0.10	-0.16	-0.14	-0.11	-0.01	0.22	0.06	0.23	0.04	0.04	**0.44**	**0.42**	1.00

Values in bold indicate moderate to strong correlations with statistical significance at *p* < 0.05.

Whereas permutation tests only yielded a significant effect of Axis 1 for Portofino, the CCA ordination tended to be similar between study areas, with some exceptions. The projection of points (perceptions) on the vectors (scuba diving experience) shows that experienced scuba divers possessed a more positive image of their underwater skills and behaviour, had more positive attitudes towards conservation, and claimed to be more knowledgeable of local conservation programmes–the latter was the case for Portofino, but not Ponta do Ouro (Fig 2). Regardless of study area, more experienced divers were less satisfied with the ecosystem’s health and management of the dive sites, and were also less critical of the negative impacts of scuba diving, whether direct, contingent, or caused by irresponsible actions (Fig 2).

**Fig 3:**
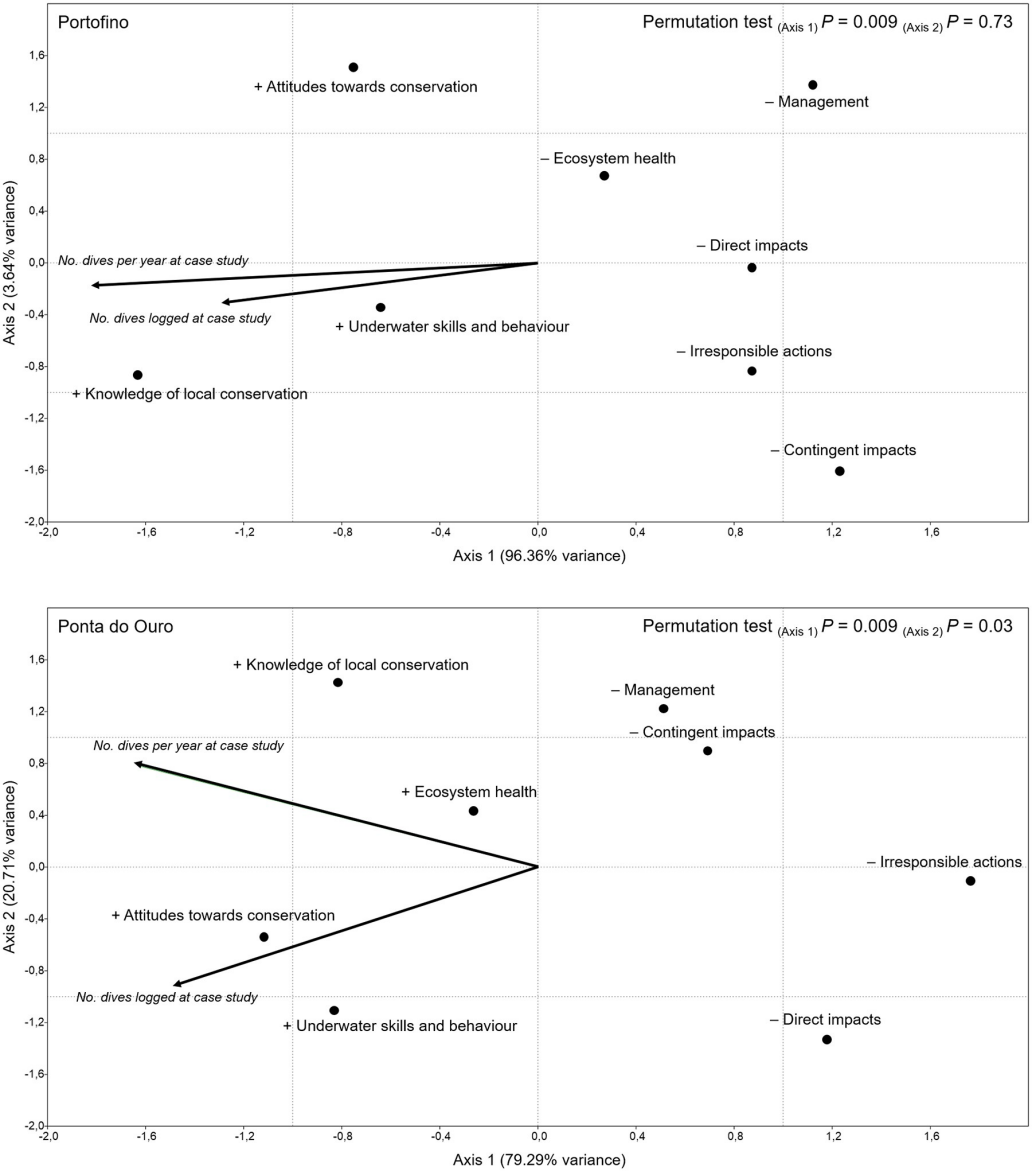
Canonical correspondence analysis (CCA) ordination biplot, displaying the influence of familiarity with the study areas (vectors) on factors of divers’ self-assessment, satisfaction with diving at the study areas, and perceptions of scuba diving impacts (points). The “+” symbol next to a factor represents a positive influence, whereas the “−” symbol represents a negative influence.

Fig 3 shows the results of the CCA determining the influence of familiarity with the study areas on divers’ perceptions. The variables describing familiarity were positively correlated (Table 3). The axes of the ordination biplot accounted for all the variation in the data. In this case, permutation tests yielded significant effects of Axis 1 for Portofino, and of both axes for Ponta do Ouro, where the CCA ordination tended to separate the total number of dives logged from annual diving frequency (Fig 3). The projections of points on vectors show that, similarly to scuba diving experience, familiarity with the study areas had a positive influence on scuba divers’ self-assessment, a negative influence on satisfaction with diving at the study areas, and a decrease in the level of damage ascribed to scuba diving impacts. However, annual diving frequency in Ponta do Ouro increased satisfaction with the ecosystem health of the dive sites, as opposed to Portofino (Fig 3).

## Discussion

### Profile of the scuba divers

The scuba divers were mostly male, middle aged and well-educated people, and skewed towards high experience and loyalty to the study areas. This profile partly reflects that of divers described in similar research carried out at both tropical [18,21,25,28,52,62–66] and non-tropical diving destinations [10,12–13,16,20,22,29–30,53,67–70], making it possible to generalise some of the implications of this study to different diving destinations.

The structure of the sample is still notably comparable between the study areas, and also longitudinally to the characteristics of the diving population of each study area according to previous literature. Divers in Portofino were more experienced than those in Ponta do Ouro. The two destinations are seemingly going through different phases in their destination lifecycles [71], with Portofino having a longer history as a scuba diving destination and a protected area than Ponta do Ouro. Comparisons with data from 13 years ago confirm that scuba divers in Portofino possess a slightly higher average level of scuba diving experience currently, and show the same degree of loyalty to the destination [42]. The scuba divers in Ponta do Ouro have become more specialised compared with data from 15 years ago [50]. Ponta do Ouro remains positioned relatively early in its lifecycle as a tourism destination [71].

### The influence of diving experience on divers’ perceptions

Diving experience affected the divers’ self-assessment positively. This finding aligns with literature demonstrating how diver specialisation is characterised by the improvement of skills and self-confidence, and by positive attitudes towards conservation [11–12,21,53].

As divers gained experience, they became less satisfied with the health of the dive sites’ ecosystem and with overall management [15,26,29,67]. In particular, satisfaction among divers in Ponta do Ouro was lower than the level reported 15 years ago [50], possibly due to the specialisation of divers in this area over time, as well as actual environmental degradation.

Experienced divers tended to view the impacts of scuba diving activities less critically than novice divers. Experienced scuba divers may become more aware of negative environmental effects of human and natural impacts other than diving [27]. They may increasingly trust their ability to self-regulate through their skills [12,15,19]. They may also deem certain harmful behaviours acceptable because they were customary in the past [13], and they possibly fear that acknowledging the potential negative impacts of diving on the environment could lead to further regulation of the activity itself.

Scuba diving experience exerted similar influences on divers, regardless of the study area. However, remarkable differences also emerged between the study areas. In Ponta do Ouro, knowledge of local conservation was not high and was only influenced by familiarity with the study area. The PPMR is a vast marine reserve (Fig 1) that was recently established and is managed through multiple efforts. Becoming acquainted with its management and conservation programmes may be challenging for tourists, in contrast to other well-established tourism destinations with a limited geographical scope (Fig 1) and less complex governance structures, for example Portofino.

Divers in Ponta do Ouro tended to appreciate the ecosystem health of the dive sites as they became more familiar with them. In contrast to Portofino, where the diving sites are delimited by fixed mooring buoys and they have relatively similar habitats (boulders, stones and vertical walls), Ponta do Ouro offers a broader variety of diving experiences, from shallow reef diving to deeper shark diving, with new dive sites being discovered over time [49]. The dive sites here are distributed over a wider area compared with Portofino, and the majority of the diving for first timers tends to happen on the closer, more popular and more crowded (and possibly less healthy) reefs. As a result, divers would need to visit more dive sites in order to become familiar with the quality of the reefs in Ponta do Ouro.

Regardless of experience, divers in Portofino tended to be more satisfied with management than those in Ponta do Ouro. This difference appears to be based on a single item, namely the presence of litter, which is a crucial issue in Ponta do Ouro [45] and has been known to detract from divers’ satisfaction [50].

Finally, divers in Ponta do Ouro underestimated the damage of direct impacts from scuba diving activities compared to Portofino divers. Specifically, the effects of walking on the sandy bottom, touching mobile wildlife accidentally, and collecting shells, pieces of coral et cetera were underestimated. Dive sites in Ponta do Ouro are reef patches surrounded by sand, and divers often adjust their buoyancy on sand before proceeding towards the reef. They are also encouraged to follow this practice during the pre-dive briefing (S. Lucrezi, pers. obs.). Interactions with mobile wildlife are very common in Ponta do Ouro, and while intentional contact with these species is highly discouraged, accidental contact is still likely to take place (E. Ferretti, pers. comm.). The bottom habitats in Ponta do Ouro have an abundance of loose coral pieces, shells and shark teeth, which are also washed on the beach by the ocean waves. The collection of this material is prohibited in Ponta do Ouro. However, many kiosks in the village sell shells and dead wildlife. This contrast is likely to create confusion among divers, who do not appear to be warned about the no-take policy in the marine reserve during the pre-dive briefing (S. Lucrezi, pers. obs.).

### Management implications of diving experience

The results of this study highlight both positive and contradictory elements related to diving experience and its influences, with implications about the persistence, but also the proper management, of a specialised diving market at a destination.

#### Enhancing the persistence of a specialised diving market

The ideal scenario for diving destinations would be to have a broad spectrum of markets, from generalists to specialists, to ensure the sustainable growth of diving tourism at the destination. Specialist divers constitute an important market, as they tend to be high-yielding, experienced, responsible, committed and proactive when it comes to conservation. Their persistence at a given destination can be ensured in three ways:

The first way involves maximising the value of the destination over the number of tourists to prevent the negative consequences of mass tourism, which is a deterrent to specialised divers. This may happen through a mixture of special marketing efforts (advertising diving tourism more than other forms of mass tourism, like beach and party tourism) and management of the tourism flow (e.g. through entry fees and traffic control) at the destination. The second way involves creating an attractive offer to a specialised market. Examples include the promotion of participatory research programmes such as monitoring and mapping and the zoning of dive destinations based on specialisation levels. The third way involves carefully planning and controlling the development of generalists in the market into specialists. This needs to happen through education, training, regulations and interpretation. Diving operators should provide high-quality ecological or research experiences to encourage diver specialisation. Generalists should also be educated on the characteristics and importance of healthy underwater ecosystems in order to avoid becoming affected by a “shifted baseline syndrome” [71].

#### Enhancing the management of a specialised diving market

The persistence of a specialised diving market also implies that this market needs to be properly managed. The divers in this study were confident in their skills and responsible behaviour, although experience influenced perceptions of the damage of diving activities negatively. There is a direct link between acknowledging the potential ecological harm of diving activities and responsible diving behaviour [9,16], and experience and self-confidence will not necessarily translate into pro-environmental behaviour [21,53,72]. Thus, the underestimation of diving impacts by experienced divers in this study can raise concern for management. Underwater photography can become a strong negative mediator in the influence of diving experience on diver behaviour [5,8]. As a result, researchers have recently proposed the introduction of courses that enable scuba divers to meet low-impact standards in underwater photography [5,66]. The results of this study support this proposition.

Other forms of management aimed at specialised divers would involve improving this market’s trust in governance. This can be achieved by voicing the concerns (especially those related to environmental degradation) of specialised divers; educating older and occasional divers on previously accepted behaviours that have become either regulated or prohibited; enhancement of personal responsibility and trust in external control by using positive messages (e.g., on the conservation benefits of regulation); and opening as well as mediating a dialogue with conflicting stakeholder groups such as fishermen.

### Implications of generalisations on diving experience

The findings of this study make it possible to formulate generalisations regarding the effects of diving experience, and to use them to manage diving activities and destinations. However, any generalisation needs to be based on an appropriate definition and measurement of scuba diving experience [10]. Furthermore, the context-specific nature of diving industries at different locations makes the use of generalisations on diving experience a delicate and, perhaps, controversial issue if the proper precautions are not taken.

Firstly, different dive locations can have very different markets and be characterised by divers with different demographic backgrounds and levels of specialisation. The use of generalisations on diving experience to justify social and environmental management actions at a destination would have to be based on data drawn from locations with comparable market structures. Secondly, diving locations are likely to follow a lifecycle as tourism destinations. Generalisations which may previously have been used for management purposes could become inapplicable after a period of time, and the monitoring of the destination’s lifecycle would become essential to assess whether new actions are needed. Thirdly, divers with similar levels of experience but who have been certified in different locations may have different impacts and views; such differences would make it impractical and risky to apply generalisations on diving experience to management decisions. Fourthly, this study compared two protected areas in which diving and other activities are regulated and controlled. This could have affected the influence of experience on divers’ perceptions and behaviour. The application of generalisations on diving experience to unprotected areas may be inappropriate when based on information drawn from studies in protected areas. Finally, the application of generalisations on diving experience for management purposes may be useless at destinations that are already naturally zoned according to market specialisation. These destinations would probably require ad hoc management actions for each zone.

## Conclusions

This study highlighted the relevance of using recreational scuba diving experience as a tool to segment scuba divers, understand their attitudes, perceptions and behaviour, and manage diving destinations accordingly. The study makes a contribution to the dearth of research assessing the potential reliability of scuba diving experience as a constant in predicting scuba diver behaviour. However, it also acknowledges the limitations resulting from making generalisations on scuba diving experience and its influence. Scuba divers represent an opportunity for the development of sustainable tourism-based livelihoods in a number of countries. It is important to ensure that research endeavours are focused on assisting the proper growth of this market, particularly the training phase, and that the findings of new research are shared with certifying agencies and governance bodies for the purpose of improving the education and management of diving activities.

## Supporting information

S1 Table Variables measured to categorise scuba divers according to experience (also as part of specialisation) and familiarity with a study area, based on examples in the literature. (RTF)

S2 Table Summary of the relevant literature about the influence of accumulated scuba diving experience on the following: Diving behaviour and personal responsibility; satisfaction and environmental perceptions; and attitudes towards conservation. (RTF)

S3 Table Questionnaire survey. (RTF)

S4 Table Descriptive statistics for items used in scaled data. (RTF)
