# Supplementary material for: The influence of scuba diving experience on divers’ perceptions, and its implications for managing diving destinations
Source: PLoS One. 2019 Jul 5;14(7):e0219306. doi: 10.1371/journal.pone.0219306 (PMC6611629; doi:10.1371/journal.pone.0219306)
Supplement: S1 Table — (RTF) [file pone.0219306.s001.rtf]

S1 Table.
Authors	Variables of scuba diving experience and specialisation**	
[1-3]	Specialisation construct based on the four social worlds of orientation, experiences, relationships and commitment; final categorisation of divers into low, moderate, high and very high specialisation	
[4]	Total number of dives logged (< 30, > 30); knowledge of marine organisms (none, basic, advanced)	
[5]	Highest scuba diving certification level; number of logged dives; number of dives logged at destination at the time of the study; membership in environmental group; reading of articles on marine life	
[6]	Certification; underwater guidance; photographic equipment; final categorisation into introduction divers, independent divers, guided divers, course divers, and photographers	
[7-8]	Specialisation construct, including behavioural (equipment, previous participation and experience), cognitive (skill level and knowledge of the activity) and affective (enduring involvement and centrality to lifestyle) items; final categorisation into the new diver, the casual diver, the active diver and the committed diver	
[9-12]	Number of logged dives	
[13]	List of all scuba diving certifications held; number of years diving; number of logged dives; year of 'marine biology/marine naturalist' certification, if any; prevalent type of diving activity	
[14]	Diving experience (amateur, skilled, professional); species identification experience (novice, intermediate, experienced)	
[15]	Highest scuba diving certification level (Not yet certified, Open/Advanced Open Water, Rescue, Divemaster, Instructor or above); number of years diving; number of logged dives; number of logged dives annually	
[16]	Diving experience rating (Beginner, Intermediate, Advanced, Expert, Post expert); number of years diving; number of logged dives	
[17]	Highest scuba diving certification level; number of logged dives	
[18]	Specialisation construct (past diving experience, investment in diving, centrality of diving to lifestyle) characterised by highest scuba diving certification level (Discover Scuba, Open Water, Advanced Open Water or higher); number of years diving; number of dives in the past two years; dive locations visited; dive gear owned; coral or fish field guides owned; final categorisation into low, medium and high specialisation	
[19]	Highest scuba diving certification level (Open Water, Advanced Open water, Higher levels)	
[20]	Highest scuba diving certification level; number of years diving; diving activity in the last 12 months (number of diving trips); preferred diving depth; familiarity with the case study (number of trips to the case study and nearby waters, number of trips to the case study and nearby waters in the last 12 months); subscription to diving magazines; membership in diving clubs; importance of diving activity among all recreational activities; diving activities sought (e.g., photography)	
[21]	Highest scuba diving certification level (Open Water, Advanced Open Water, Specialty, Master Diver, Divemaster, Instructor, Master Instructor); number of years diving; number of logged dives; familiarity with the case study (whether or not this was the first trip to the case study)	
[22]	Highest scuba diving certification level (Discover scuba, Open water, Advanced Open Water, Rescue, Divemaster, Instructor); certification location; number of logged dives annually (< 10 for novice, 11-30 for intermediate, 31-50 for experienced, > 50 for very experienced); days elapsed since last dive	
[23]	Highest scuba diving certification level; number of years diving; number of logged dives; final categorisation into beginner, intermediate, advanced, professional	
[24]	Number of years diving (< 10, 10-19, 20 or above); familiarity with the case study (number of years diving at the case study)	
[25]	Highest scuba diving certification level (Open Water, Divemaster, Instructor, Trainee); number of logged dives (< 100 for novice, 100 or above for experienced); number of years diving; familiarity with the case study (number of previous trips to the case study)	
[26]	Highest scuba diving certification level (Open Water, Advanced Open Water, Divemaster, Master Instructor); number of logged dives; familiarity with the case study (number of logged dives at the case study)	
[27]	Highest scuba diving certification level (beginner, intermediate, expert); number of years diving; number of logged dives	
[28]	Highest scuba diving certification level; membership in scuba diving club; number of years diving; familiarity with the case study (number of previous trips to the case study)	
[29]	Number of years diving; total number of dive trips; highest scuba diving certification level (Open Water, Advanced Open Water, Divemaster, Instructor); diver level (beginner, intermediate, expert); places dived (ocean, freshwater, wreck, cave); number of countries dived; number of logged dives annually	
[30]	Highest scuba diving certification level (Supervised diver, Autonomous diver, Dive leader, Instructor level I, Instructor level II); number of years diving; number of logged dives; final categorisation into groups with little experience, moderate experience and much experience	
[31]	Specialisation construct characterised by behavioural (number of logged dives) and cognitive (highest scuba diving certification level, self-rated experience) components	
[32]	Highest scuba diving certification level (Open Water, Advanced Open Water, Rescue); number of years diving; number of logged dives; self-categorisation (beginner, intermediate, advanced); final categorisation into learners, dabblers, enthusiasts	
[33]	Dive certification agency; highest scuba diving certification level (Open Water, Advanced Open Water, Rescue, Divemaster, Instructor, Other); number of years diving; familiarity with the case study (number of times dived at the case study)	
[34]	Highest scuba diving certification level (Open Water, Advanced Open Water, Divemaster); number of logged dives	
[35]	Highest scuba diving certification level (None, Open Water, Advanced Open Water, Master Diver, Rescue Diver, Divemaster or higher); number of years diving; number of logged dives; ownership of scuba diving equipment; travelling for the exclusive purpose of diving; familiarity with the case study (number of lifetime logged dives at the case study; number of previous trips to the case study)	
[36]	Highest scuba diving certification level (None, Open Water, Advanced Open Water, Master Diver, Rescue Diver, Divemaster or higher); number of years diving; number of logged dives; ownership of scuba diving equipment; travelling for the exclusive purpose of diving; subscription to diving magazine; membership in diving or marine conservation club; familiarity with the case study (number of previous trips to the case study)	
[37]	Highest scuba diving certification level (recreational, professional); number of logged dives (< 50, 51-500, > 500)	
[38]	Highest scuba diving certification level; number of years diving; number of days diving; number of logged dives; self-categorisation into new diver, casual diver, active diver, committed diver	
[39]	Specialisation construct of self-classification into casual, active and committed diver. Construct included number of days diving in the last year; number of dives in the last year; number of years diving; self-reported skill level compared to other divers; highest scuba diving certification level (Open Water, Advanced Open Water, Rescue, Master diver, Divemaster, Professional); number of scuba diving specialties held; importance of scuba diving activity; membership in diving club or organisation; diving magazine subscription; estimated cost of owned scuba diving equipment; commitment to scuba diving	
[40]	Highest scuba diving certification level (Open Water, Advanced Open Water, Divemaster or above); number of dives logged; number of years diving	
[41-42]	Full specialisation construct, including behavioural (experience and frequency of participation, familiarity with the case study), cognitive (certification level, self-reported skill level) and affective (enduring involvement, centrality to lifestyle) items	
[43]	Full specialisation construct, including diving experience, skill, participation, knowledge, equipment, commitment, amateur/professional growth, self-categorisation (beginner, intermediate, advanced, expert, post-expert)	
[44]	Full specialisation construct, including diving experience, self-categorisation (beginner, intermediate, advanced, expert, post-expert), diving motivations, diving in the previous year, constraining factors, diving expenditures	
[45]	Highest scuba diving certification level (Open Water, Advanced Open Water, Rescue, Divemaster, Instructor); number of dives logged	
[46]	Number of logged dives (1-10 for novice, 11-50 for moderately experienced, > 50 for very experienced); days elapsed since last dive	
[47]	Highest scuba diving certification level (recreational, professional); number of logged dives (< 20 for inexperienced recreational, > 20 for experienced recreational, > 500 for professional)	
[48]	Number of logged dives (1-25 for beginners, 26-50 for novices, 51-100 for enthusiasts, > 100 for experts)	
** Categories are provided in brackets for categorised variables.	

References
1.	Anderson LE, Loomis DK. Scuba diver specialization and behavior norms at coral reefs. Coast Manage. 2011;39(5): 478-491.
2.	Paterson S, Young S, Loomis DK, Obenour W. Resource attributes that contribute to nonresident diver satisfaction in the Florida Keys, USA. Tourism Mar Environ. 2012;8(1-2): 47-60.
3.	Young S, Loomis D. Diver perceptions of Florida Keys Reef conditions by specialization level. In: Watts CE Jr., Fisher CL, editors. Proceedings of the 2009 Northeastern recreation research symposium. Newtown Square, PA: United States Department of Agriculture, Forest Service; 2010. pp. 24-29.
4.	Arvanitidis C, Faulwetter S, Chatzigeorgiou G, Penev L, Bánki O, Dailianis T, et al. Engaging the broader community in biodiversity research: the concept of the COMBER pilot project for divers in ViBRANT. ZooKeys. 2011;150: 211-229.
5.	Barker NH, Roberts CM. Scuba diver behaviour and the management of diving impacts on coral reefs. Biol Conserv. 2004;120(4): 481-489.
6.	Belhassen Y, Rousseau M, Tynyakov J, Shashar N. Evaluating the attractiveness and effectiveness of artificial coral reefs as a recreational ecosystem service. J Environ Manage. 2017;203: 448-456.
7.	Bentz J, Rodrigues A, Dearden P, Calado H, Lopes F. Crowding in marine environments: divers and whale watchers in the Azores. Ocean Coast Manage. 2015;109: 77-85.
8.	Bentz J, Lopes F, Calado H, Dearden P. Managing marine wildlife tourism activities: analysis of motivations and specialization levels of divers and whale watchers. Tour Manage Perspect. 2016;18: 74-83.
9.	Camp E, Fraser D. Influence of conservation education dive briefings as a management tool on the timing and nature of recreational SCUBA diving impacts on coral reefs. Ocean Coast Manage. 2012;61: 30-37.
10.	Giglio VJ, Luiz OJ, Chadwick NE, Ferreira CE. Using an educational video-briefing to mitigate the ecological impacts of scuba diving. J Sust Tour. 2018;26(5): 782-797.
11.	Roberts L, Harriott VJ. Recreational scuba diving and its potential for environmental impact in a marine reserve. In Bellwood O, Choat H, Saxena N, editors. Recent Advances in Marine Science and Technology. Townsville, Qld: PACON International and James Cook University of North Queensland; 1994. pp. 695-704.
12.	Zhang L, Chung S. Assessing the social carrying capacity of diving sites in Mabul Island, Malaysia. Environ Manage. 2015;56(6): 1467-1477.
13.	Cerrano C, Milanese M, Ponti M. Diving for science – science for diving: volunteer scuba divers support science and conservation in the Mediterranean Sea. Aquat Conserv. 2017;27(2): 303–323.
14.	Chatzigeorgiou G, Faulwetter S, Dailianis T, Smith VS, Koulouri P, Dounas C, et al. Testing the robustness of Citizen Science projects: evaluating the results of pilot project COMBER. Biodivers Data J. 2016;4: e10859.
15.	Chung SS, Au A, Qiu JW. Understanding the underwater behaviour of scuba divers in Hong Kong. Environ Manage. 2013;51(4): 824-837.
16.	Cottrell SP, Meisel C. Predictors of personal responsibility to protect the marine environment among scuba divers. In: Murdy J, editor. Proceedings of the 2003 Northeastern recreation research symposium. Newtown Square, PA: United States Department of Agriculture, Forest Service; 2004. pp. 252-261.
17.	De Brauwer M, Saunders BJ, Ambo-Rappe R, Jompa J, McIlwain JL, Harvey ES. Time to stop mucking around? Impacts of underwater photography on cryptobenthic fauna found in soft sediment habitats. J Environ Manage. 2018;218: 14-22.
18.	Dearden P, Bennett M, Rollins R. Implications for coral reef conservation of diver specialization. Environ Conserv. 2006;33(4): 353-363.
19.	Di Franco A, Milazzo M, Baiata P, Tomasello A, Chemello R. Scuba diver behaviour and its effects on the biota of a Mediterranean marine protected area. Environ Conserv. 2009;36(1): 32-40.
20.	Ditton RB, Osburn HR, Baker TL, Thailing CE. Demographics, attitudes, and reef management preferences of sport divers in offshore Texas waters. ICES J Mar Sci. 2002;59: S186-S191.
21.	Edney J. Diver characteristics, motivations, and attitudes: Chuuk Lagoon. Tourism Mar Environ. 2012;8(1-2): 7-18.
22.	Hammerton Z. Determining the variables that influence SCUBA diving impacts in eastern Australian marine parks. Ocean Coast Manage. 2017;142: 209-217.
23.	Hammerton Z, Dimmock K, Hahn C, Dalton SJ, Smith SD. Scuba diving and marine conservation: collaboration at two Australian subtropical destinations. Tourism Mar Environ. 2012;8(1-2): 77-90.
24.	Johnson AE, Jackson JB. Fisher and diver perceptions of coral reef degradation and implications for sustainable management. Glob Ecol Conserv. 2015;3: 890-899.
25.	Kirkbride-Smith AE, Wheeler PM, Johnson ML. The relationship between diver experience levels and perceptions of attractiveness of artificial reefs-examination of a potential management tool. PLoS One. 2013;8(7): e68899.
26.	Lucrezi S, Saayman M, Van der Merwe P. Managing diving impacts on reef ecosystems: Analysis of putative influences of motivations, marine life preferences and experience on divers' environmental perceptions. Ocean Coast Manage. 2013;76: 52-63.
27.	Luna B, Pérez CV, Sánchez-Lizaso JL. Benthic impacts of recreational divers in a Mediterranean Marine Protected Area. ICES J Mar Sci. 2009;66(3): 517-523.
28.	Mundet L, Ribera L. Characteristics of divers at a Spanish resort. Tourism Manage. 2011;22(5): 501-510.
29.	Musa G, Seng WT, Thirumoorthi T, Abessi M. The influence of scuba divers' personality, experience, and demographic profile on their underwater behavior. Tourism Mar Environ. 2011;7(1): 1-14.
30.	Neto AQ, Lohmann G, Scott N, Dimmock K. Rethinking competitiveness: important attributes for a successful scuba diving destination. Tour Rec Res. 2017;42(3): 356-366.
31.	Ong TF, Musa G. Examining the influences of experience, personality and attitude on SCUBA divers' underwater behaviour: a structural equation model. Tourism Manage. 2012;33(6): 1521-1534.
32.	Pabel A, Coghlan A. Dive market segments and destination competitiveness: a case study of the Great Barrier Reef in view of changing reef ecosystem health. Tourism Mar Environ. 2011;7(2): 55-66. 
33.	Roche RC, Harvey CV, Harvey JJ, Kavanagh AP, McDonald M, Stein-Rostaing VR, et al. Recreational diving impacts on coral reefs and the adoption of environmentally responsible practices within the SCUBA diving industry. Environ Manage. 2016;58(1): 107-116.
34.	Rouphael AB, Inglis GJ. “Take only photographs and leave only footprints”?: an experimental study of the impacts of underwater photographers on coral reef dive sites. Biol Conserv. 2001;100(3): 281-287.
35.	Schuhmann PW, Casey JF, Horrocks JA, Oxenford HA. Recreational SCUBA divers' willingness to pay for marine biodiversity in Barbados. J Environ Manage. 2013;121: 29-36.
36.	Schuhmann PW, Cazabon-Mannette M, Gill D, Casey JF, Hailey A. Willingness to pay to avoid high encounter levels at dive sites in the Caribbean. Tourism Mar Environ. 2013;9(1-2): 81-94.
37.	Smith KR, Scarpaci C, Scarr MJ, Otway NM. Scuba diving tourism with critically endangered grey nurse sharks (Carcharias taurus) off eastern Australia: tourist demographics, shark behaviour and diver compliance. Tourism Manage. 2014;45: 211-225.
38.	Sorice MG, Oh CO, Ditton RB. Managing scuba divers to meet ecological goals for coral reef conservation. Ambio. 2007;36(4): 316-322.
39.	Sorice MG, Oh CO, Ditton RB. Exploring level of support for management restrictions using a self-classification measure of recreation specialization. Leis Sci. 2009;31(2): 107-123.
40.	Szuster BW, Needham MD, McClure BP. Scuba diver perceptions and evaluations of crowding underwater. Tourism Mar Environ. 2011;7(3-4): 153-165.
41.	Thapa B, Graefe AR, Meyer LA. Moderator and mediator effects of scuba diving specialization on marine-based environmental knowledge-behavior contingency. J Environ Educ. 2005;37(1): 53-67.
42.	Thapa B, Graefe AR, Meyer LA. Specialization and marine based environmental behaviors among SCUBA divers. J Leisure Res. 2006;38(4): 601-615.
43.	Todd SL, Cooper T, Graefe AR. Scuba diving & underwater cultural resources: differences in environmental beliefs, ascriptions of responsibility, and management preferences based on level of development. In: Kyle G, editor. Proceedings of the 2000 Northeastern recreation research symposium. Newtown Square, PA: United States Department of Agriculture, Forest Service; 2001. pp. 131-140. 
44.	Todd SL, Graefe AR, Mann W. Differences in SCUBA diver motivations based on level of development. In: Todd S, editor. Proceedings of the 2001 Northeastern recreation research symposium. Newtown Square, PA: United States Department of Agriculture, Forest Service; 2002. pp. 107-114.
45.	Toyoshima J, Nadaoka K. Importance of environmental briefing and buoyancy control on reducing negative impacts of SCUBA diving on coral reefs. Ocean Coast Manage. 2015;116: 20-26.
46.	Walters RDM, Samways MJ. Sustainable dive ecotourism on a South African coral reef. Biodivers Conserv. 2001;10(12): 2167-2179.
47.	Ward-Paige CA, Lotze HK. Assessing the value of recreational divers for censusing elasmobranchs. PLoS One. 2011;6(10): e25609.
48.	Worachananant S, Carter RW, Hockings M, Reopanichkul P. Managing the impacts of SCUBA divers on Thailand's coral reefs. J Sust Tour. 2008;16(6): 645-663.
