# Supplementary material for: The influence of scuba diving experience on divers’ perceptions, and its implications for managing diving destinations
Source: PLoS One. 2019 Jul 5;14(7):e0219306. doi: 10.1371/journal.pone.0219306 (PMC6611629; doi:10.1371/journal.pone.0219306)
Supplement: S2 Table — (RTF) [file pone.0219306.s002.rtf]

S2 Table.
Theme	Type of study	Outcomes	Interpretations	Authors	
Diving behaviour and personal responsibility	Mostly observation of underwater diver behaviour and assessment of damage to habitats, sometimes (but not necessarily) associated with questionnaire- or interview-based surveys. A relatively small fraction of study is only based on surveys. 	There is an inverted relationship between experience and damage. Experience is linked to a higher sense of responsibility towards the marine environment (e.g., litter removal, awareness of the consequences of negative diving behaviour, support to businesses adhering to the standards of environmental responsibility).	Experienced divers are better able to control their buoyancy compared to novice divers; they also have a regular commitment to the scuba diving activity sustaining the levels of underwater comfort. 	[1-14]	
		There is a positive relationship between diving experience and direct damage to bottom habitats.	Experienced divers use specialist equipment (e.g., cameras) and engage in activities that may cause substantial harm to underwater habitats when unregulated.	[2,13,15-29]	
			Novice divers can be more cautious due to lower confidence, thus inflicting less ecological harm. Damage by experienced divers can be related to sensitive habitats such as caves and wrecks, which are accessible only to this group, and to situations in which challenging conditions may lead to more contact. Experienced divers accept contact behaviour if done “correctly”.	[2,7,21,30]	
			Even for the same certifications, effectiveness of training may differ significantly across locations and dive shops, making it difficult to draw relationships between experience and damage caused.	[2]	
		There is no relationship between diving experience and direct damage to bottom habitats.	Under the influence of environmentally responsible diving programmes due to stern regulations, zoning and underwater intervention, the potential relationship between diving experience and environmental harm can be neutralised in protected areas.	[13,16,23]	
		There is a mixed relationship between diving experience and personal responsibility.	Factors such as knowledge of environmental issues, environmental beliefs and environmental concern may have a greater influence on personal responsibility than diving experience. Diver specialisation does not necessarily affect social norms equally.	[1,5,31-32]	
Satisfaction and environmental perceptions	Mostly surveys to assess the limits of acceptable change at diving destinations or satisfaction (diver preferences, expectations, perceived environmental health of dive sites, perceived crowding and encountered norms at dive sites, and overall satisfaction with the diving experience), finalised for better management or marketing. 	There is an inverted relationship between experience and satisfaction, as well as between experience and tolerance of crowding or environmental conditions, perceived as diminished.	The results are consistent with theories of recreation specialisation whereby experience is linked to increasing environmental knowledge, attachment to the resource the activity depends on, a willingness to see such resources unharmed and unexploited, and preferences for conditions that will not spoil the quality of diving.	[33-47]	
			Experienced and older divers have local expert knowledge, have possibly visited many other locations and are better placed to perceive and quantify changes. They are willing to abandon a location as a result of perceived environmental degradation.	[27,36,48-51]	
			Novice divers tend to derive comfort from being surrounded by people underwater and to prioritise attributes of the dive that are mostly related to safety, visibility and accessibility. They are possibly more accepting of alternative diving settings such as artificial reefs. Contemporary novice divers may be affected by the so-called “shifting baseline syndrome”, whereby new generations are gradually accepting increasingly degraded seascapes.	[35,37-40,44,49-50,52]	
		There is no relationship between diving experience and either preferences or satisfaction.	There is an absence of observed relationship and a limited number of variables used to describe diving experience.	[33,48,53]	
Attitudes towards conservation	Mostly surveys to assess the level of support for management and conservation (including a willingness to pay for research).	Experienced divers can fully comply with established codes of conduct and are supportive of new rules and conservation-oriented regulations.	Experienced divers tend to embrace an environmental ethos and desire to learn and contribute to problem-solving through active participation in science. They are also a high-yielding market and are financially able to support conservation of the resources their activity depends on.	[48,54-56]	
		Experienced divers tend to be less dependent upon guidance and in favour of flexible norms.	Experienced divers may be less willing to invest in the protection of diving locations, as they are able to visit many other alternative ones. Based on negative past experiences, they may also distrust managerial interventions.	[35,54,57-59]	
		Experienced divers oppose increased diving rates.	An unwillingness to pay may be due to the diminished 'marginal utility' (in economics, the satisfaction or benefit derived from consuming a product) of diving with increasing experience.	[60]	
		Novice divers can be more accepting of different types of norms, regardless of their severity.	Novice divers probably have no choice but to pay in order to protect the fewer locations accessible to them, and are possibly willing to pay for diver fees due to their limited experience of diving without paying.	[57-59,61-62]	

References
1.	Anderson LE, Loomis DK. Scuba diver specialization and behavior norms at coral reefs. Coast Manage. 2011;39(5): 478-491.
2.	Hammerton Z. Determining the variables that influence SCUBA diving impacts in eastern Australian marine parks. Ocean Coast Manage. 2017;142: 209-217.
3.	Lucrezi S, Saayman M, Van der Merwe P. Managing diving impacts on reef ecosystems: Analysis of putative influences of motivations, marine life preferences and experience on divers' environmental perceptions. Ocean Coast Manage. 2013;76: 52-63.
4.	Luna B, Pérez CV, Sánchez-Lizaso JL. Benthic impacts of recreational divers in a Mediterranean Marine Protected Area. ICES J Mar Sci. 2009;66(3): 517-523.
5.	Musa G, Seng WT, Thirumoorthi T, Abessi M. The influence of scuba divers' personality, experience, and demographic profile on their underwater behavior. Tourism Mar Environ. 2011;7(1): 1-14.
6.	Thapa B, Graefe AR, Meyer LA. Moderator and mediator effects of scuba diving specialization on marine-based environmental knowledge-behavior contingency. J Environ Educ. 2005;37(1): 53-67.
7.	Thapa B, Graefe AR, Meyer LA. Specialization and marine based environmental behaviors among SCUBA divers. J Leisure Res. 2006;38(4): 601-615.
8.	Toyoshima J, Nadaoka K. Importance of environmental briefing and buoyancy control on reducing negative impacts of SCUBA diving on coral reefs. Ocean Coast Manage. 2015;116: 20-26.
9.	Walters RDM, Samways MJ. Sustainable dive ecotourism on a South African coral reef. Biodivers Conserv. 2001;10(12): 2167-2179.
10.	Worachananant S, Carter RW, Hockings M, Reopanichkul P. Managing the impacts of SCUBA divers on Thailand's coral reefs. J Sust Tour. 2008;16(6): 645-663.
11.	Bravo G, Márquez F, Marzinelli EM, Mendez MM, Bigatti G. Effect of recreational diving on Patagonian rocky reefs. Mar Environ Res. 2015;104: 31-36.
12.	Heyman, WD, Carr LM, Lobel PS. Diver ecotourism and disturbance to reef fish spawning aggregations: it is better to be disturbed than to be dead. Mar Ecol Prog Ser. 2010;419: 201-210.
13.	Krieger JR, Chadwick NE. Recreational diving impacts and the use of pre-dive briefings as a management strategy on Florida coral reefs. J Coast Conserv. 2013;17(1): 179-189.
14.	Zakai D, Chadwick-Furman NE. Impacts of intensive recreational diving on reef corals at Eilat, northern Red Sea. Biol Conserv. 2002;105(2): 179-187.
15.	Barker NH, Roberts CM. Scuba diver behaviour and the management of diving impacts on coral reefs. Biol Conserv. 2004;120(4): 481-489.
16.	Belhassen Y, Rousseau M, Tynyakov J, Shashar N. Evaluating the attractiveness and effectiveness of artificial coral reefs as a recreational ecosystem service. J Environ Manage. 2017;203: 448-456.
17.	Camp E, Fraser D. Influence of conservation education dive briefings as a management tool on the timing and nature of recreational SCUBA diving impacts on coral reefs. Ocean Coast Manage. 2012;61: 30-37.
18.	Giglio VJ, Luiz OJ, Chadwick NE, Ferreira CE. Using an educational video-briefing to mitigate the ecological impacts of scuba diving. J Sust Tour. 2018;26(5): 782-797.
19.	Chung SS, Au A, Qiu JW. Understanding the underwater behaviour of scuba divers in Hong Kong. Environ Manage. 2013;51(4): 824-837.
20.	De Brauwer M, Saunders BJ, Ambo-Rappe R, Jompa J, McIlwain JL, Harvey ES. Time to stop mucking around? Impacts of underwater photography on cryptobenthic fauna found in soft sediment habitats. J Environ Manage. 2018;218: 14-22.
21.	Di Franco A, Milazzo M, Baiata P, Tomasello A, Chemello R. Scuba diver behaviour and its effects on the biota of a Mediterranean marine protected area. Environ Conserv. 2009;36(1): 32-40.
22.	Ong TF, Musa G. Examining the influences of experience, personality and attitude on SCUBA divers' underwater behaviour: a structural equation model. Tourism Manage. 2012;33(6): 1521-1534.
23.	Roche RC, Harvey CV, Harvey JJ, Kavanagh AP, McDonald M, Stein-Rostaing VR, et al. Recreational diving impacts on coral reefs and the adoption of environmentally responsible practices within the SCUBA diving industry. Environ Manage. 2016;58(1): 107-116.
24.	Hammerton Z, Bucher D. Levels of intervention–reducing SCUBA-diver impact within subtropical marine protected areas. J Ecotour. 2015;14(1): 3-20.
25.	Medio D, Ormond RFG, Pearson M. Effect of briefings on rates of damage to corals by scuba divers. Biol Conserv. 1997;79(1): 91-95.
26.	Harriott VJ, Davis D, Banks SA. Recreational diving and its impact in marine protected areas in eastern Australia. Ambio. 1997;26(3): 173-179.
27.	Pereira MAM. Recreational SCUBA diving and reef conservation in southern Mozambique. M.Sc. Thesis, University of Natal. 2003.
28.	Rouphael AB, Inglis GJ. Impacts of recreational scuba diving at sites with different reef topographies. Biol Conserv. 1997;82(3): 329-336.
29.	Uyarra MC, Côté IM. The quest for cryptic creatures: impacts of species-focused recreational diving on corals. Biol Conserv. 2007;136(1): 77-84.
30.	Rouphael AB, Inglis GJ. “Take only photographs and leave only footprints”?: an experimental study of the impacts of underwater photographers on coral reef dive sites. Biol Conserv. 2001;100(3): 281-287.
31.	Cottrell SP, Meisel C. Predictors of personal responsibility to protect the marine environment among scuba divers. In: Murdy J, editor. Proceedings of the 2003 Northeastern recreation research symposium. Newtown Square, PA: United States Department of Agriculture, Forest Service; 2004. pp. 252-261.
32.	Ong TF, Musa G. SCUBA divers' underwater responsible behaviour: can environmental concern and divers' attitude make a difference?. Curr Issues Tour. 2012;15(4): 329-351.
33.	Young S, Loomis D. Diver perceptions of Florida Keys Reef conditions by specialization level. In: Watts CE Jr., Fisher CL, editors. Proceedings of the 2009 Northeastern recreation research symposium. Newtown Square, PA: United States Department of Agriculture, Forest Service; 2010. pp. 24-29.
34.	Bentz J, Rodrigues A, Dearden P, Calado H, Lopes F. Crowding in marine environments: divers and whale watchers in the Azores. Ocean Coast Manage. 2015;109: 77-85.
35.	Dearden P, Bennett M, Rollins R. Implications for coral reef conservation of diver specialization. Environ Conserv. 2006;33(4): 353-363.
36.	Johnson AE, Jackson JB. Fisher and diver perceptions of coral reef degradation and implications for sustainable management. Glob Ecol Conserv. 2015;3: 890-899.
37.	Kirkbride-Smith AE, Wheeler PM, Johnson ML. The relationship between diver experience levels and perceptions of attractiveness of artificial reefs-examination of a potential management tool. PLoS One. 2013;8(7): e68899.
38.	Neto AQ, Lohmann G, Scott N, Dimmock K. Rethinking competitiveness: important attributes for a successful scuba diving destination. Tour Rec Res. 2017;42(3): 356-366.
39.	Schuhmann PW, Casey JF, Horrocks JA, Oxenford HA. Recreational SCUBA divers' willingness to pay for marine biodiversity in Barbados. J Environ Manage. 2013;121: 29-36.
40.	Schuhmann PW, Cazabon-Mannette M, Gill D, Casey JF, Hailey A. Willingness to pay to avoid high encounter levels at dive sites in the Caribbean. Tourism Mar Environ. 2013;9(1-2): 81-94.
41.	Sorice MG, Oh CO, Ditton RB. Managing scuba divers to meet ecological goals for coral reef conservation. Ambio. 2007;36(4): 316-322.
42.	Szuster BW, Needham MD, McClure BP. Scuba diver perceptions and evaluations of crowding underwater. Tourism Mar Environ. 2011;7(3-4): 153-165.
43.	Anderson LE, Loomis DK. Normative standards for coral reef conditions: a comparison of SCUBA divers by specialization level. J Leisure Res. 2012;44(2): 257-274. 
44.	Inglis GJ, Johnson VI, Ponte F. Crowding norms in marine settings: a case study of snorkeling on the Great Barrier Reef. Environ Manage. 1999;24(3): 369-381.
45.	Catlin J, Jones R. Whale shark tourism at Ningaloo Marine Park: a longitudinal study of wildlife tourism. Tourism Manage. 2010;31(3): 386-394.
46.	Leujak W, Ormond RF. Visitor perceptions and the shifting social carrying capacity of South Sinai's coral reefs. Environ Manage. 2007;39(4): 472-489.
47.	Meisel-Lusby C, Cottrell S. Understanding motivations and expectations of scuba divers. Tourism Mar Environ. 2008;5(1): 1-14. 
48.	Bentz J, Lopes F, Calado H, Dearden P. Managing marine wildlife tourism activities: analysis of motivations and specialization levels of divers and whale watchers. Tour Manage Perspect. 2016;18: 74-83.
49.	Pabel A, Coghlan A. Dive market segments and destination competitiveness: a case study of the Great Barrier Reef in view of changing reef ecosystem health. Tourism Mar Environ. 2011;7(2): 55-66. 
50.	Augustine S, Dearden P, Rollins R. Are changing diver characteristics important for coral reef conservation?. Aquat Conserv. 2016;26(4): 660-673.
51.	Loerzel JL, Goedeke TL, Dillard MK, Brown G. SCUBA divers above the waterline: using participatory mapping of coral reef conditions to inform reef management. Mar Policy. 2017;76: 79-89.
52.	Paterson S, Young S, Loomis DK, Obenour W. Resource attributes that contribute to nonresident diver satisfaction in the Florida Keys, USA. Tourism Mar Environ. 2012;8(1-2): 47-60.
53.	Zhang L, Chung S. Assessing the social carrying capacity of diving sites in Mabul Island, Malaysia. Environ Manage. 2015;56(6): 1467-1477.
54.	Edney J. Diver characteristics, motivations, and attitudes: Chuuk Lagoon. Tourism Mar Environ. 2012;8(1-2): 7-18.
55.	Hammerton Z, Dimmock K, Hahn C, Dalton SJ, Smith SD. Scuba diving and marine conservation: collaboration at two Australian subtropical destinations. Tourism Mar Environ. 2012;8(1-2): 77-90.
56.	Smith KR, Scarpaci C, Scarr MJ, Otway NM. Scuba diving tourism with critically endangered grey nurse sharks (Carcharias taurus) off eastern Australia: tourist demographics, shark behaviour and diver compliance. Tourism Manage. 2014;45: 211-225.
57.	Mundet L, Ribera L. Characteristics of divers at a Spanish resort. Tourism Manage. 2011;22(5): 501-510.
58.	Sorice MG, Oh CO, Ditton RB. Exploring level of support for management restrictions using a self-classification measure of recreation specialization. Leis Sci. 2009;31(2): 107-123.
59.	Todd SL, Cooper T, Graefe AR. Scuba diving & underwater cultural resources: differences in environmental beliefs, ascriptions of responsibility, and management preferences based on level of development. In: Kyle G, editor. Proceedings of the 2000 Northeastern recreation research symposium. Newtown Square, PA: United States Department of Agriculture, Forest Service; 2001. pp. 131-140. 
60.	Asafu-Adjaye J, Tapsuwan S. A contingent valuation study of scuba diving benefits: case study in Mu Ko Similan Marine National Park, Thailand. Tourism Manage. 2008;29(6): 1122-1130.
61.	Roberts M, Hanley N, Cresswell W. User fees across ecosystem boundaries: are SCUBA divers willing to pay for terrestrial biodiversity conservation?. J Environ Manage. 2017;200: 53-59.
62.	Uyarra MC, Gill JA, Côté IM. Charging for nature: marine park fees and management from a user perspective. Ambio. 2010;39(7): 515-523.
