# Supplementary material for: The influence of scuba diving experience on divers’ perceptions, and its implications for managing diving destinations
Source: PLoS One. 2019 Jul 5;14(7):e0219306. doi: 10.1371/journal.pone.0219306 (PMC6611629; doi:10.1371/journal.pone.0219306)
Supplement: S3 Table — (RTF) [file pone.0219306.s003.rtf]

S3 Table.
SECTION A
1. Gender:				M	1	F	2			5. Marital status:													
												 	 																					
2. Year of birth:					 	 	 	 	 	 			Single	 	 	 	 	 	 	 	 	 	 	 	 	 	1	
																		Married	 	 	 	 	 	 	 	 	 	 	 	 	 	2	
3. Education:														De facto	 	 	 	 	 	 	 	 	 	 	 	 	 	3	
																		Divorced	 	 	 	 	 	 	 	 	 	 	 	 	4	
No school	 	 	 	 	 	 	 	 	 	 	 	1		Widow/er	 	 	 	 	 	 	 	 	 	 	 	 	5	
Matric	 	 	 	 	 	 	 	 	 	 	 	 	2																				
Diploma or professional	 	 	 	 	 	 	3		6. Occupation:													
Graduate	 	 	 	 	 	 	 	 	 	 	 	4																				
Master's degree	 	 	 	 	 	 	 	 	 	5		Student	 	 	 	 	 	 	 	 	 	 	 	 	 	1	
PhD or equivalent	 	 	 	 	 	 	 	 	6		Paid work	 	 	 	 	 	 	 	 	 	 	 	 	2	
Other (specify):	 	 	 	 	 	 	 	 	 	7		Unpaid work	 	 	 	 	 	 	 	 	 	 	 	3	
 	 	 	 	 	 	 	 	 	 	 	 	 	 	 				Unemployed	 	 	 	 	 	 	 	 	 	 	 	4	
																		Retired	 	 	 	 	 	 	 	 	 	 	 	 	 	5	
3.1. If your answer to question 3 was 3, 4, 5, 6, 																					
or 7, what was your main subject?							6.1. If you are/have been employed,						
 	 	 	 	 	 	 	 	 	 	 	 	 	 	 	 	 		what is/was your main profession?						
																		 	 	 	 	 	 	 	 	 	 	 	 	 	 	 	 	 	 	
																																				
4.Which country are you from?								
 	 	 	 	 	 	 	 	 	 	 	 	 	 	 	 	 		
																		


7. How many certifications do you hold in each of the following?															
																																Number	
A. Basic (Open, Advanced, Rescue)	 	 	 	 	 	 	 	 	 	 	 	 	 	 	 	 	 	 	 	
B. Pro (Dive Master, Assistant Instructor/Instructor of any level or speciality)	 	 	 	 	 	 	 	
C. Speciality (caves/caverns, ice, recreational nitrox, but not	 	 	 	 	 	 	 	 	 	 	 	 	
     "technical" and "dry" below)	 	 	 	 	 	 	 	 	 	 	 	 	 	 	 	 	 	 	 	 	 		
D. Tecnical (all types/levels for Trimix, Rebreather, Side Mount, Deco 	 	 	 	 	 	 	 	 	
     Dive/Decompression, DPV etc.)	 	 	 	 	 	 	 	 	 	 	 	 	 	 	 	 	 	 	 		
E. Dry (Oxygen provider, Gas blender,	 	 	 	 	 	 	 	 	 	 	 	 	 	 	 	 	 	 	 	
     Equipment specialist, First aid, Boat operator etc.)	 	 	 	 	 	 	 	 	 	 	 	 	 		
F. Anything that cannot be listed in the above or that you are unsure of:	 	 	 	 	 	 	 	
 	 	 	 	 	 	 	 	 	 	 	 	 	 	 	 	 	 	 	 	 	 	 	 	 	 	 	 	 	 	 	 	 	 	 	 	
																																				
8. Which certifying agency, among those that issued your certifications, represents you the most?		
		 	 	 	 	 	 	 	 	 	 	 	 	 	 	 	 	 	 	 	 	 	 	 	 	 	 	 	 	 	 	 	 	 	 	
																																				
9. What year did you start diving?							11. On average, how many dives do you log per year?	
 	 	 	 	 	 	 	 	 	 	 	 	 	 	 	 	 		 	 	 	 	 	 	 	 	 	 	 	 	 	 	 	 	 	 	
																																				
10. How many dives have you logged (TOT)?			12. Do you take photos while you dive here?	
 	 	 	 	 	 	 	 	 	 	 	 	 	 	 	 	 															YES	NO	

13. Is it the first time you dive at this destination?				14. How many dives have you logged at this destination?	
													YES	NO											 	 	 	 	 	 	 	 	 	
																																				
15. If you are a repeat visitor, how many times do you dive at this destination per year?	
																											 	 	 	 	 	 	 	 	 	


SECTION B
1. Please indicate to what extent you agree with the following statements:																
Strongly agree	
Agree	 	
Neutral		 	
Disagree	 		 	
Strongly disagree	 	 	 	 	
1. I know all the local diving regulations.	1	2	3	4	5	
2. While diving, I keep neutrally buoyant at all times.	1	2	3	4	5	
3. I know what marine conservation programmes are run locally.	1	2	3	4	5	
4. I keep a good distance from the sea bottom when I dive. 	1	2	3	4	5	
5. I know there are fines for breaking local diving regulations.	1	2	3	4	5	
6. I know what pre-diving procedures have to be followed locally.	1	2	3	4	5	
7. I possess the necessary skills to dive at this destination.	1	2	3	4	5	
8. I know how to use underwater diving equipment.	1	2	3	4	5	
9. I reproach divers who do not pay attention to pre-dive briefings.	1	2	3	4	5	
10. I am or want to be involved in local marine conservation.	1	2	3	4	5	
11. I would like to be involved in the general local conservation.	1	2	3	4	5	
12. I practice good finning technique when I dive.	1	2	3	4	5	
13. When I dive, I observe wildlife quietly without chasing it.	1	2	3	4	5	
14. I reproach divers who break underwater rules.	1	2	3	4	5	


2. What is your level of satisfaction with the following aspects of your diving experience at this destination?	
																																				
 	 	 	 	 	 	 	 	 	 	 	 	 	 	 	 	 	 	 	 	 	 	 	 	 	 	 	 	 	 	 	 	 	Satisfied	
 	 	 	 	 	 	 	 	 	 	 	 	 	 	 	 	 	 	 	 	 	 	 	 	 	 	 	 	 	 	 	 	 Neutral	 	
 	 	 	 	 	 	 	 	 	 	 	 	 	 	 	 	 	 	 	 	 	 	 	 	 	 	 	 	 	 	 	Unsatisfied	 		
a. Water cleanliness	1	2	3	
b. Abundance of marine life	1	2	3	
c. Litter	1	2	3	
d. Visibility	1	2	3	
e. Local diving regulations	1	2	3	
f. Variety of small species (e.g., nudibranchs)	1	2	3	
g. Variety of sessile life (e.g., sponges, coral, gorgonians)	1	2	3	
h. The general health of the dive sites	1	2	3	
i. The variety of big species (e.g., grouper)	1	2	3	
j. The crowding of dive sites	1	2	3	
k. The underwater conduct of fellow divers	1	2	3	
l. The pre-dive briefing	1	2	3	
m. The conduct of the divemaster	1	2	3	

3. In your opinion, what is the damage of the following diving aspects to the bottom habitats?				
Heavy	
Moderate		
Little	 		
None	 	 	 	
a. Walking on the sandy bottom before or during a dive	1	2	3	4	
b. Diving with gloves	1	2	3	4	
c. Touching mobile wildlife (e.g., fish, octopuses, turtles) on purpose	1	2	3	4	
d. Collecting shells, pieces of coral or other	1	2	3	4	
e. Spear fishing	1	2	3	4	
f. Flash photography	1	2	3	4	
g. Being a novice diver	1	2	3	4	
h. Anchoring the boat before a dive	1	2	3	4	
i. Touching sessile wildlife (e.g., sponges, gorgonians) on purpose	1	2	3	4	
j. Drift diving	1	2	3	4	
k. Diving alone and not with a buddy/group	1	2	3	4	
l. Videotaping underwater	1	2	3	4	
m. Diving at night	1	2	3	4	
n. A bad pre-dive briefing	1	2	3	4	
o. Losing or leaving gear behind underwater	1	2	3	4	
p. Touching sessile wildlife (e.g., sponges, gorgonians) accidentally	1	2	3	4	
q. Wearing sunscreen before a dive	1	2	3	4	
r. Not diving with an operator or charter	1	2	3	4	
s. Achieving buoyancy near or on the bottom	1	2	3	4	
t. Noise from boats on the surface	1	2	3	4	
u. Touching mobile wildlife (e.g., fish, octopuses, turtles) accidentally	1	2	3	4	
v. Using special equipment/configuration (e.g., side mount)	1	2	3	4	
w. Drinking and diving	1	2	3	4	
x. Chasing or standing in the way of mobile wildlife (e.g., fish, turtles)	1	2	3	4	
