# Supplementary material for: The influence of scuba diving experience on divers’ perceptions, and its implications for managing diving destinations
Source: PLoS One. 2019 Jul 5;14(7):e0219306. doi: 10.1371/journal.pone.0219306 (PMC6611629; doi:10.1371/journal.pone.0219306)
Supplement: S4 Table — (RTF) [file pone.0219306.s004.rtf]

S4 Table.
	Valid N	Mean	Minimum	Maximum	Standard deviation	Standard error	Skewness	Kurtosis	
I know all local diving regulations.	485	3,64	1	5	1,08	0,05	-0,54	-0,33	
I keep neutrally buoyant at all times while diving.	486	4,10	1	5	0,90	0,04	-0,88	0,49	
I know the marine conservation programmes that are run locally.	484	3,11	1	5	1,22	0,06	-0,03	-0,88	
I keep a good distance from the bottom habitats while diving.	486	3,98	1	5	0,88	0,04	-0,63	0,02	
I know what fines are given for breaking local diving regulations.	484	3,60	1	5	1,28	0,06	-0,61	-0,68	
I know what pre-diving procedures have to be followed locally.	482	3,80	1	5	1,03	0,05	-0,70	-0,02	
I possess the necessary skills to dive locally.	482	4,24	1	5	0,82	0,04	-1,14	1,52	
I know how to use scuba diving equipment.	486	4,49	1	5	0,68	0,03	-1,43	2,75	
I reproach divers who do not pay attention to the pre-dive briefings.	482	3,54	1	5	1,15	0,05	-0,38	-0,66	
I am or want to be involved in local marine conservation.	480	3,60	1	5	1,12	0,05	-0,42	-0,45	
I would like to be involved in local conservation.	481	3,56	1	5	1,10	0,05	-0,33	-0,48	
I practice good finning technique when I dive.	485	4,20	1	5	0,80	0,04	-0,90	0,84	
I observe wildlife quietly without chasing it when I dive.	482	4,48	1	5	0,69	0,03	-1,43	2,82	
I reproach divers who break underwater rules.	480	3,80	1	5	1,09	0,05	-0,63	-0,27	
Water cleanliness	472	2,75	1	3	0,48	0,02	-1,66	1,86	
Abundance of marine life	479	2,73	1	3	0,51	0,02	-1,76	2,26	
Litter	467	2,36	1	3	0,77	0,04	-0,70	-0,96	
Visibility	474	2,50	1	3	0,56	0,03	-0,55	-0,74	
Local diving regulations	466	2,44	1	3	0,63	0,03	-0,69	-0,51	
Variety of small species	474	2,47	1	3	0,63	0,03	-0,78	-0,41	
Variety of coral and sessile life	474	2,61	1	3	0,57	0,03	-1,09	0,20	
The general health of the dive sites	475	2,53	1	3	0,60	0,03	-0,91	-0,17	
Variety of big species	473	2,59	1	3	0,60	0,03	-1,21	0,41	
Crowding of dive sites	472	2,35	1	3	0,61	0,03	-0,39	-0,66	
The underwater conduct of fellow divers	470	2,46	1	3	0,63	0,03	-0,76	-0,43	
The pre-dive briefing	474	2,66	1	3	0,56	0,03	-1,41	1,03	
The conduct of the divemaster	471	2,72	1	3	0,54	0,02	-1,83	2,42	
Walking on the sandy bottom before or during a dive	479	2,54	1	4	1,07	0,05	-0,01	-1,24	
Diving with gloves	465	2,03	1	4	0,94	0,04	0,49	-0,76	
Purposefully touching mobile wildlife	482	3,28	1	4	0,92	0,04	-1,00	-0,14	
Collecting shells, pieces of coral or other	483	3,22	1	4	1,05	0,05	-1,01	-0,41	
Spear fishing	480	3,30	1	4	1,00	0,05	-1,12	-0,12	
Flash photography	476	2,21	1	4	0,88	0,04	0,23	-0,71	
Being a novice diver	469	2,43	1	4	0,87	0,04	0,12	-0,65	
Anchoring the boat before a dive	474	3,13	1	4	1,01	0,05	-0,83	-0,54	
Purposefully touching sessile wildlife	479	3,41	1	4	0,83	0,04	-1,34	1,03	
Drift diving	467	1,86	1	4	0,88	0,04	0,70	-0,40	
Diving alone and not with a buddy/group	472	2,46	1	4	1,27	0,06	0,08	-1,67	
Videotaping underwater	471	1,45	1	4	0,72	0,03	1,50	1,50	
Diving at night	471	1,69	1	4	0,82	0,04	1,02	0,33	
A bad pre-dive briefing	473	2,86	1	4	0,96	0,04	-0,37	-0,88	
Losing or leaving gear behind underwater	474	2,87	1	4	0,97	0,04	-0,30	-1,02	
Touching sessile wildlife accidentally	476	3,07	1	4	0,93	0,04	-0,64	-0,59	
Wearing sunscreen before a dive	470	2,30	1	4	1,03	0,05	0,28	-1,06	
Not diving with an operator or charter	469	2,47	1	4	1,07	0,05	0,08	-1,24	
Achieving buoyancy near or on the bottom	471	2,69	1	4	0,94	0,04	-0,26	-0,80	
Noise from boats on the surface	475	2,48	1	4	0,87	0,04	0,04	-0,66	
Touching mobile wildlife accidentally	477	2,89	1	4	0,94	0,04	-0,30	-0,98	
Using special equipment/configurations	455	1,71	1	4	0,81	0,04	0,93	0,16	
Drinking and diving	481	3,19	1	4	1,03	0,05	-1,00	-0,30	
Chasing or standing in the way of mobile wildlife	482	3,07	1	4	0,99	0,04	-0,70	-0,67	
